# Influence of public external debt on government health expenditure: A mixed-methods case study of Senegal

**DOI:** 10.1371/journal.pgph.0006698

**Published:** 2026-07-10

**Authors:** Frederik Federspiel, Josephine Borghi, Henning Tarp Jensen, Elhadji Mamadou Mbaye, Melisa Martinez Alvarez

**Affiliations:** 1 Department of Global Health and Development, London School of Hygiene and Tropical Medicine, London, United Kingdom; 2 Health, Ageing and Health Systems Research Group, International Institute for Applied Systems Analysis, Laxenburg, Austria; 3 Department of Food and Resource Economics, University of Copenhagen, Copenhagen, Denmark; 4 Department of Political Science, Gaston Berger University, Saint Louis, Senegal; 5 Institut de Recherche en Santé, de Surveillance Epidemiologique et de Formation (IRESSEF), Dakar, Senegal; 6 Independent Consultant; School of Public Health, College of Health Science, Addis Ababa University, ETHIOPIA

## Abstract

Public external debt burdens of low- and middle-income countries have grown substantially over the past decade linked to increased borrowing. Our empirical understanding of how external official loans and their ensuing debt burden may impact government health spending at the individual country level is limited. We aimed to better understand these relationships through a case study of Senegal. We conducted a mixed-methods case study using descriptive quantitative analyses of loan, debt and health financing statistics in Senegal from 1970-2023. We also used the Framework method to thematically analyse semi-structured interview data collected between October 2019 and January 2020 from 25 key informants from government ministries, development partners, academia, civil society and hospital management in Senegal. We triangulated these data with findings from a purposive document review of reports from government websites and academic literature discussing loan and debt effects on government health expenditure in Senegal. Debt obligations resulting from external loans appear to have had a constraining effect on the government budget including for health. Development loans have however also supported the health sector directly. We found less support for an indirect benefit to the health budget through loans to other sectors, due to substantial decreases in government health expenditure out of general government expenditure from 2000-2022. Overall, our findings indicate that Senegal’s debt burden has constrained government health expenditure, serving as a possible co-determinant of the regression seen on the Abuja pledge since 2000. To mitigate the negative effect of debt repayment, official external creditors should consider more flexible debt repayment timelines and exploring options for debt relief including debt-to-health swaps. Supported by debt relief, increased government priority of the health sector working toward the Abuja pledge will be necessary to make progress on universal health coverage. We encourage further country level research documenting debt impacts on health financing.

## Introduction

Across Low- and Middle-Income Countries (LMICs), external official creditors provide loans to recipient governments as general budget support or earmarked funds to specific sectors, including the health sector. From the perspective of the health sector in a given country, loans may thereby help grow the health budget directly through lending for health, or indirectly through loans stimulating economic growth and government revenue raising, which can in turn be allocated for health purposes. Over time however, debt plus its interest accumulates and needs repayment from the general government budget, which can affect the fiscal space available for sectoral budget allocations - including for health [1–5]. This results in a question of relative balance: Which sectors benefit more from development lending, and which sectors are “picking up the tab” for repaying investments in other sectors?

**Fig 1:**
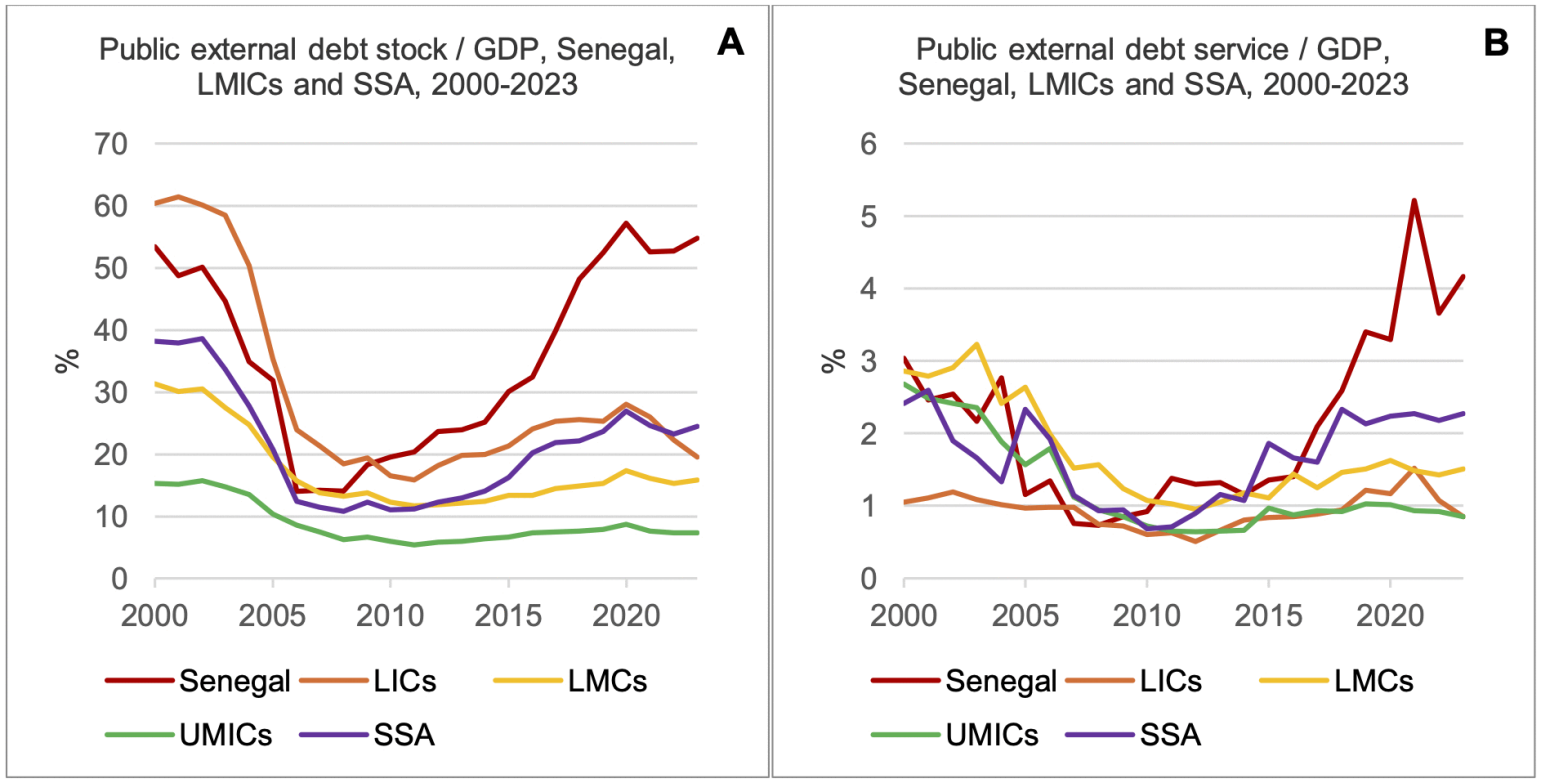
Public and Publicly Guaranteed (PPG) external debt stocks (panel A) and service (panel B) as fractions of Gross Domestic Product (GDP) in Senegal compared to Low- and Middle-Income Countries (LMICs) and Sub-Saharan Africa (SSA) (%), 2000-2023 [8]. SSA grouping is with high income countries excluded. LICs: Low Income Countries. LMCs: Lower Middle-income Countries. UMICs: Upper Middle-Income Countries.

In the late 1990’s and early 2000’s, the public external debt burdens of LMICs were relatively high: average Public and Publicly Guaranteed (PPG) external debt stocks hovered around 20% of Gross Domestic Product (GDP) and PPG external debt servicing levels around 2.5% of GDP, with great variation between countries, regions and income groups (Fig 1). This was viewed as unsustainable for many countries, constricting fiscal space in turn limiting investments in social sectors. Debt relief was granted to help countries free up resources to develop and fund their hard-pressed education and health sectors [6,7]. This resulted in falling debt obligations during the 2000s [8–11]. The debt burdens of LMICs to foreign creditors have since been growing over the following decade, as debt relief decreased and public external borrowing increased [12] (Fig 1). In 2022, 49 LMICs spent more on public external debt servicing than on health compared to 33 in 2012 (out of 112 LMICs with data available for both years) [13,14]. Across LMICs, real public debt interest payments per capita surged during the COVID-19 pandemic, with further increases projected up until 2029 especially in Upper-Middle-Income Countries (UMICs) [15]. Recent calls have been made for debt resolution, including by the United Nations and the WB, to ensure funding can be sustained for public sectors, including health sectors, and the Sustainable Development Goals (SDGs) in general (e.g., [16–25]).

Existing LMIC literature has examined the association between public debt and government health spending through quantitative cross-country panel data analyses in African [1,26–29], Latin American [2], South- and Southeast Asian [4], and select “emerging” and middle-income countries [30] as well as across LMICs overall [3,31,32]. We were only able to identify two single-country case studies, both from Kenya, that used respectively mixed and quantitative methods to examine links between the country’s debt burden and government health spending [33,34]. The majority of the identified studies find negative associations between debt burdens and government health spending. This is similar to most of the identified econometric literature on public debt and education spending, mostly finding a negative link [26,27,35–41]. For health, this indicates a challenge for the path to Universal Health Coverage (UHC) [42].

The existing quantitative, cross-sectional, multi-country analyses are limited in their ability to assess individual country budget realities, prioritisations made and the underlying reasons for this. As seen, there are limited country case studies of the impacts of debt obligations on government health spending in LMICs. With global debt burdens growing, understanding individual country realities as they balance financial obligations to their external creditors and to their health sectors is increasingly important. How do governments prioritise, are they able to protect the health sector from debt obligations, if so how, and is the health sector equally impacted as for example the education sector? What policy lessons can be learnt from these individual country experiences? Sub-Saharan Africa (SSA) in particular is vulnerable with relatively low levels of health spending, growing debt burdens, and a relatively low resilience to economic shocks [13,14]. On an indebted continent, our case country of Senegal is among the most indebted [8], allowing for a highly topical exploration of these questions under conditions of a high debt burden, with potential lessons for other countries on a similar track. At 3.3%, its level of domestic government health spending (Government Health Spending as a Source, GHE-S) out of General Government Expenditure (GGE) also ranked as number 44 out of 49 SSA countries with data in 2022 [14]. Even when including external transfers distributed by the government (on-budget development assistance for the health sector), bringing government health expenditure to 4.9% of GGE, this is still far from the pledge made by African Union countries in 2001 as part of the Abuja declaration to spend 15% of government budgets on the health sector [14,43]. Our study aims to understand the influence of external official loans and the ensuing debt obligations on GHE-S in the context of an indebted SSA country, by performing an in-depth mixed-methods case study of Senegal [14,44].

## Materials and methods

### Study setting

**Fig 2:**
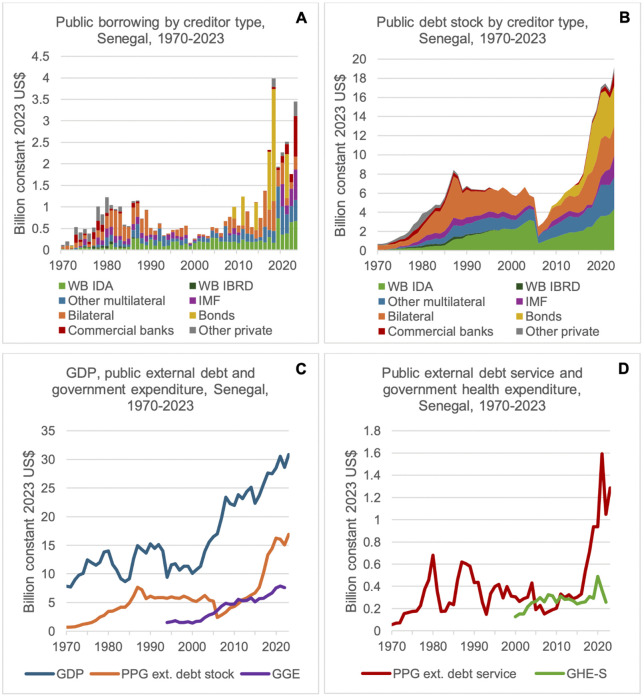
Panel A: Public and Publicly Guaranteed (PPG) borrowing in Senegal separated by creditor type, 1970-2023 (billion constant 2023 US$) [8,12]. **Panel B**: PPG debt stock in Senegal from 1970-2023 [12]. **Panel C**: Gross Domestic Product (GDP) (1970-2023), PPG external debt stock (1970-2023), and General Government Expenditure (GGE) in Senegal (1994-2022) [8,49]. **Panel D**: PPG external debt service (1970-2023) and Government Health Expenditure as a Source (GHE-S) (2000-2022) in Senegal [8,14]. Please see S1 Appendix for additional details on the numbers used to produce this figure.

Situated in Francophone West Africa, Senegal has been one of the most stable democracies in the region, and in Africa more broadly [45], except for political turmoil that erupted over former President Macky Sall’s attempt to postpone the 2024 presidential elections [46]. The country became independent from French colonial rule in 1960 [45]. In 2023, the country had a population of approximately 18 million [47] and a GDP per capita of $1,706 in 2023 [8], resulting in a WB classification as a Lower Middle-income Country (LMC). We chose Senegal as a case country for four main reasons. A: Its political stability at the time of fieldwork, so our results were reflective of creditor-government dynamics under stable conditions. B: Its relatively low perceived corruption levels (2023 and earlier) to minimise the risk of changes in health financing occurring due to corruption and not the factors of interest to our study [48]. C: A growing public external debt burden [8] (Fig 1 and Fig 2). D: A health financing composition increasingly characterized by user fees and less so by government contributions at the time our interviews were performed in 2019/20 [14] (Fig 5), inviting questions as to the determinants of this trend.

S1 Appendix contains additional information on the health system organisation in Senegal.

### Study approach

This study was a mixed-methods case study comprised of descriptive statistical analysis of health financing contributions in Senegal, key stakeholder interviews and purposive document review. The analysis of quantitative data and document analysis were performed after the initial analysis of interview data, going back and forth between all sources of data while writing up the results.

We considered the following as evidence of external official lending and public external debt obligations influencing government health spending. A: Verbal statements from interviewees identifying a such influence or effect; B: written documentation of a such, or C: discernible patterns between levels and changes in external official lending, public external debt servicing and government health spending.

The following sections describe our three different methods applied.

### Quantitative data collection and analysis

We assembled publicly available secondary data on PPG loan disbursements in Senegal separated by creditor type and PPG external debt stock and service from the WB Open Data and International Debt Statistics databases [8,12]. General government revenue and expenditure data were obtained from the IMF Public Finances in Modern History and WB Open Data databases [8,49]; total official development loans received by sector from the OECD Creditor Reporting System [50]; and GHE-S, External health financing (EXT), OOP and Voluntary Health Insurance (VHI) from the World Health Organization (WHO) Global Health Expenditure Database [14].

Data was used as historically available in databases. WB data on public borrowing and debt were available from 1970-2023; IMF/WB data on government revenue and expenditure from 1994-2022; OECD Official Development Assistance (ODA) data from 2002-2023; and WHO data on health financing from 2000-2022. All dollar-values were deflated into 2023 constant US$ using WB US$ GDP deflator data [8].

We analysed and presented the quantitative data as trends over time, comparing changes in public borrowing, debt burden and the levels and balance of different health financing sources over time, as well as the overall sectoral distribution of development lending in Senegal. This was done to A: examine the extent to which the health sector has benefited from development loans; B: explore associations between debt servicing levels and government health spending, and C: add context to and triangulate findings from other sources. This was not done to directly establish causality, but rather to generate hypotheses and look for evidence supporting or challenging findings from other data sources.

For this analysis, the general hypothesis was applied of an inverse relationship between debt servicing and government health spending due to a general budget constraint. For comparison, we also explored levels and changes in government education spending to look for evidence of debt servicing impacting across social sectors or differentially between them, using data from the WB [8].

### Qualitative data collection and analysis

#### Interviews.

To identify potential key informants, we first purposively mapped stakeholders in Senegal with roles relevant to health financing and macro-fiscal policy based on author knowledge and searching relevant organisation websites. We then began contacting identified individuals and initiated interviews, and then used snowball sampling to identify further key informants. A total of 32 key informants were identified for interview, of which 25 agreed to participate. Our final sample included representatives of four bilateral development partners, four multilateral development partners, 9 from central government across four different ministries, four from Senegalese civil society organisations, three from academia and one from hospital management.

Interviews were conducted in Dakar between September 30, 2019, to January 31, 2020, by FF, who was a PhD student at the time. He had completed a course on Semi-Structured Interviews (SSIs) at LSHTM before conducting interviews. He was supervised by MMA, who had extensive experience with SSIs before this study. The SSI method was used to allow the interviewer to probe and explore interviewee responses in further details as relevant. Our interview topic guide was tested through conducting a preliminary mock interview with an external researcher, who provided critical feedback on both content and interview style, which was integrated before commencing stakeholder interviews. Interviews were conducted in French or English, using an interpreter for interviews in French. Interviews were recorded and transcribed by a professional transcriptionist, where consent was given for this. The participant information sheet used, consent form and an example interview topic guide can be found in the online LSHTM data repository [51] and S1 Appendix.

The interview data were analysed using the Framework method [52]. Themes were established deductively based on the research questions as well as inductively from the interviews. The thematic coding framework was validated by an independent researcher before coding all interview data, using NVivo 12 [53]. Participant views and key quotes were summarised across themes in a Framework matrix [52,54]. Identified patterns in these summary findings were then integrated into the results section. All interviewee statements were anonymised when writing up the paper.

#### Document review.

We searched Senegalese government websites [55–60], and the Senegalese websites of the International Monetary Fund (IMF) [61,62] and the World Bank (WB) [63] for documents discussing an influence of public external lending on government health spending (215 full text documents reviewed). We chose to review documents from the IMF and WB due to the central, privileged positions of these institutions in monitoring, analysing and advising on fiscal policy, as summarised in numerous staff reports, strategy papers and more.

We also searched across all major academic publishing databases, using the London School of Hygiene & Tropical Medicine Discover platform and Google Scholar, looking for academic literature describing external official lending or public external debt in Senegal, including its effects on health spending (no time constraint, see S1 Appendix for search strings used). Ten full-text academic articles were retrieved for review. Our inclusion criteria for the results section was that documents/articles discussed effects of lending on government health spending in Senegal. The document review was carried out by FF, who read through the documents while applying our inclusion criteria and integrating relevant findings into the results section in stages from the time interviews were conducted in 2019/20 up until March 2025.

### Ethics statement

We obtained ethical approval from the London School of Hygiene Observational/ Interventions Research Ethics Committee (*ref: 16420*) and the National Health Research Ethics Committee in Senegal (*protocol SEN19/56, ref:* 00172). Formal written consent was obtained from all study participants.

## Results

### Trends in borrowing and debt

In this section, we analyse Senegal’s borrowing and debt stock and servicing levels over time in relation to GDP and government expenditure, including on health.

Between 1970–2023, Senegal has borrowed approximately US$48.2 billion from its creditors (constant 2023 US$. *Note: The used IMF lending data includes drawings made on the IMF general resource account, not the reserve tranche, for which we were unable to find a data source. IMF disbursements and resulting outstanding credit are not counted in PPG external debt figures and were added to attain the aggregate numbers presented in this section*) [12]. Of this, approximately US$12.2 billion has been borrowed from bilateral creditors, US$9.6 billion from the WB’s concessional IDA branch, US$8.7 billion from other multilateral creditors, US$6.6 billion via bonds, US$5.9 billion from commercial banks and other private creditors, US$4.8 billion from the IMF, and $0.4 billion from the WB International Bank for Reconstruction and Development (IBRD) branch (*note: Some of the amount borrowed via bonds may be double counted in the other categories, as these creditor types may buy government bonds, however we were unable to identify more detailed data on the holders of Senegalese bonds to be able to avoid this*) [8].

As shown in Fig 2, Senegalese public borrowing increased during the 1970’s, leading to debt buildup, which was partly relieved in the Heavily Indebted Poor Countries initiative, providing 850 million US$ in debt relief from 2000-2004 [64]. Since 2000, Senegalese annual borrowing levels from foreign creditors have risen substantially, up from approximately $0.3 billion in 2000 to $3.5 billion in 2023 (constant 2023 US$) [8]. Increases were most pronounced from 2016 and onward, in part due to the government’s issuance of US$5.4 billion worth of bonds (Eurobonds) in separate tranches, beginning in 2009 [8,65–67] (Fig 2). By 2023, Senegal owed a total US$19.2 billion to its creditors [8]. Of this, $4.2 billion was owed to bondholders, US$4.4 billion to the WB IDA, $3.3 billion to other multilateral creditors, $3.1 billion to bilateral creditors, $2.3 billion to the IMF, and $2.0 billion to commercial banks and other private creditors [8,12] (Fig 2).

The PPG external debt of Senegal corresponded to 55% of its GDP in 2023, well above the average for other LMCs (16%) and SSA countries (25%) (Fig 1) [8]. In 2021, real-term PPG external debt servicing peaked at US$1.6 billion (constant 2023 US$), corresponding to 20% of GGE, outgrowing GHE-S by a factor of 4.3 [8,14,49]. While having subsequently decreased, it has done so in proportion to GHE-S which also decreased, maintaining a factor of 4.1 compared to GHE-S in 2022 (Fig 2).

### Has lending directly benefited the health sector?

**Fig 3:**
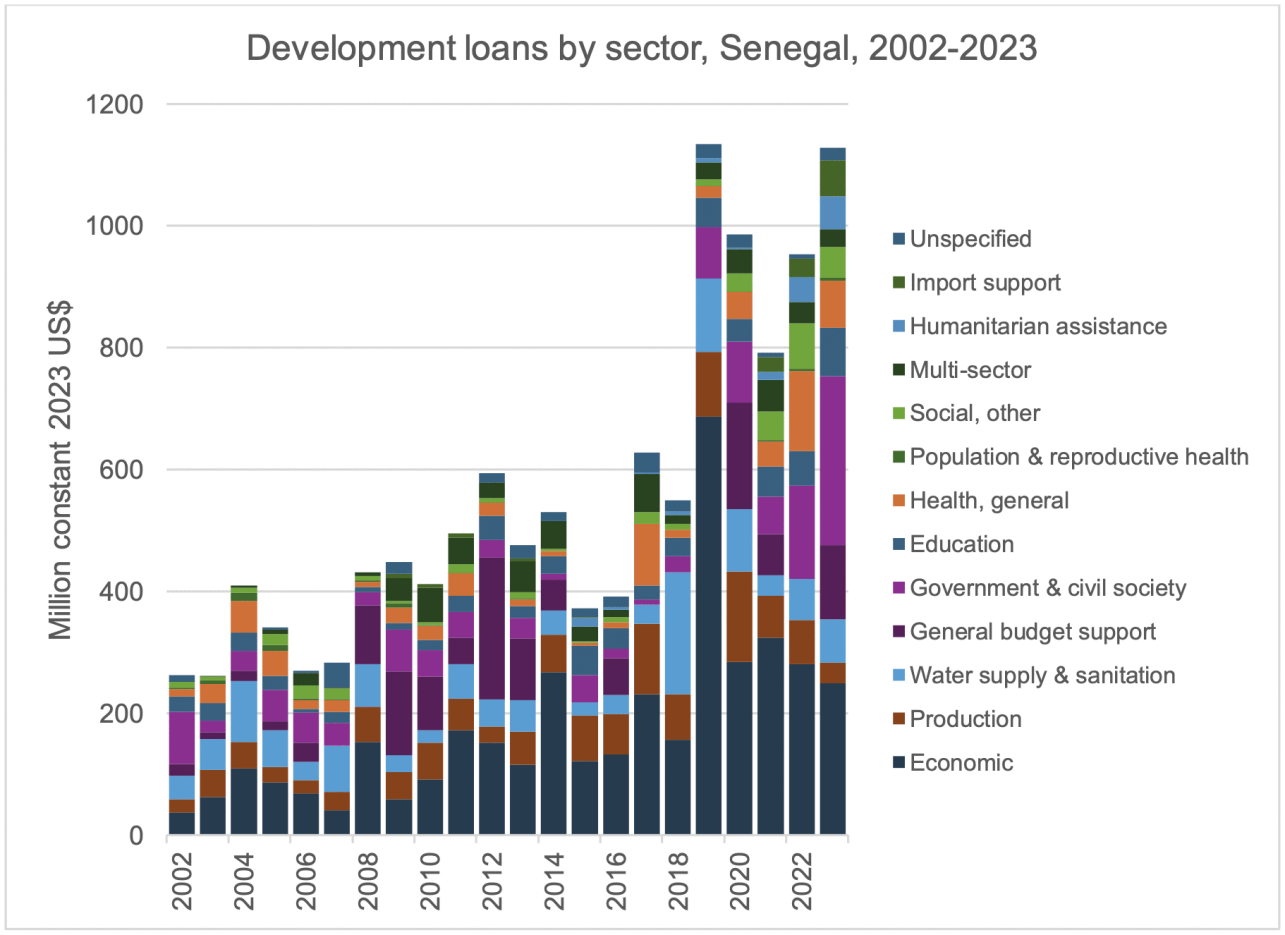
Total official development loans received by sector, Senegal, 2002-23 [50]. Data deflated using US$ GDP deflator [8].

Fig 3 shows the distribution of development loans received in Senegal between 2002–23 by sector [50]. Health and population and reproductive health have received approximately 7% of all development loans, compared to 32% to economic sectors, 11% to production sectors, and 6% to the education sector [50].

Although not the primary focus of ODA lending, the observation can be made that direct benefit to the health sector from ODA lending has increased over time. This has occurred as a growing amount of ODA has been disbursed for health (Fig 5), of which a growing share has been disbursed as loans [50] (S1 Appendix). In 2023, 33% of ODA for health was disbursed as loans, compared to 3% in 2015, following a general trend for ODA across all sectors in Senegal [50] (S1 Appendix). This expansion has not occurred at the expense of ODA grants for health, which were US$170 million in 2015 and US$163 million in 2023 (constant 2023 US$) [50].

Several interviewees across stakeholder groups however correctly believed that external official loans predominantly went to economically profitable sectors (natural resources, production, manufacturing etc.), and not social sectors. The government and donors were explained as being jointly interested in ensuring a return on investment:

*“I think lending goes more into the economic sectors, and the health and social sectors are less important… The donors or partners who finance … expect a return on investment. They have not invested much in the social, the social is not economically profitable, so many partners prefer to invest in the economic than in the social.”* (CSO)

A government interviewee concurred that they encouraged external official loans where these could be profitable:

*“… Now more and more, we still want to guide partners to finance loans especially in areas that can be profitable”.* (Government)

### Has lending indirectly benefited the health sector?

**Fig 4:**
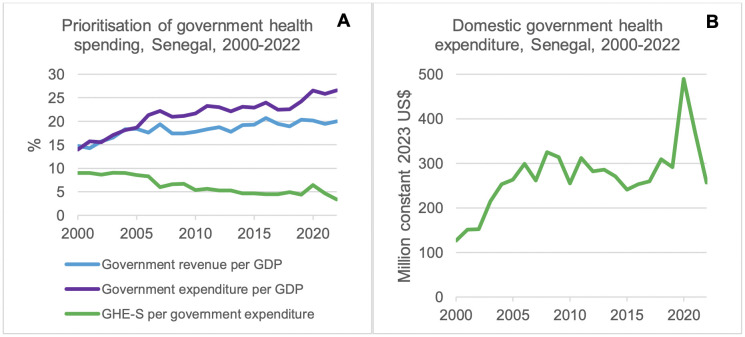
Panel A: General Government Expenditure (GGE) and government revenue as a proportion of GDP and Government Health Expenditure as a Source (GHE-S) as a proportion of GGE in Senegal between 2000-2022 [14,49]. **Panel B**: GHE-S from 2000-2022 in Senegal [14]. Data deflated using WB US$ GDP deflator [8].

**Fig 5:**
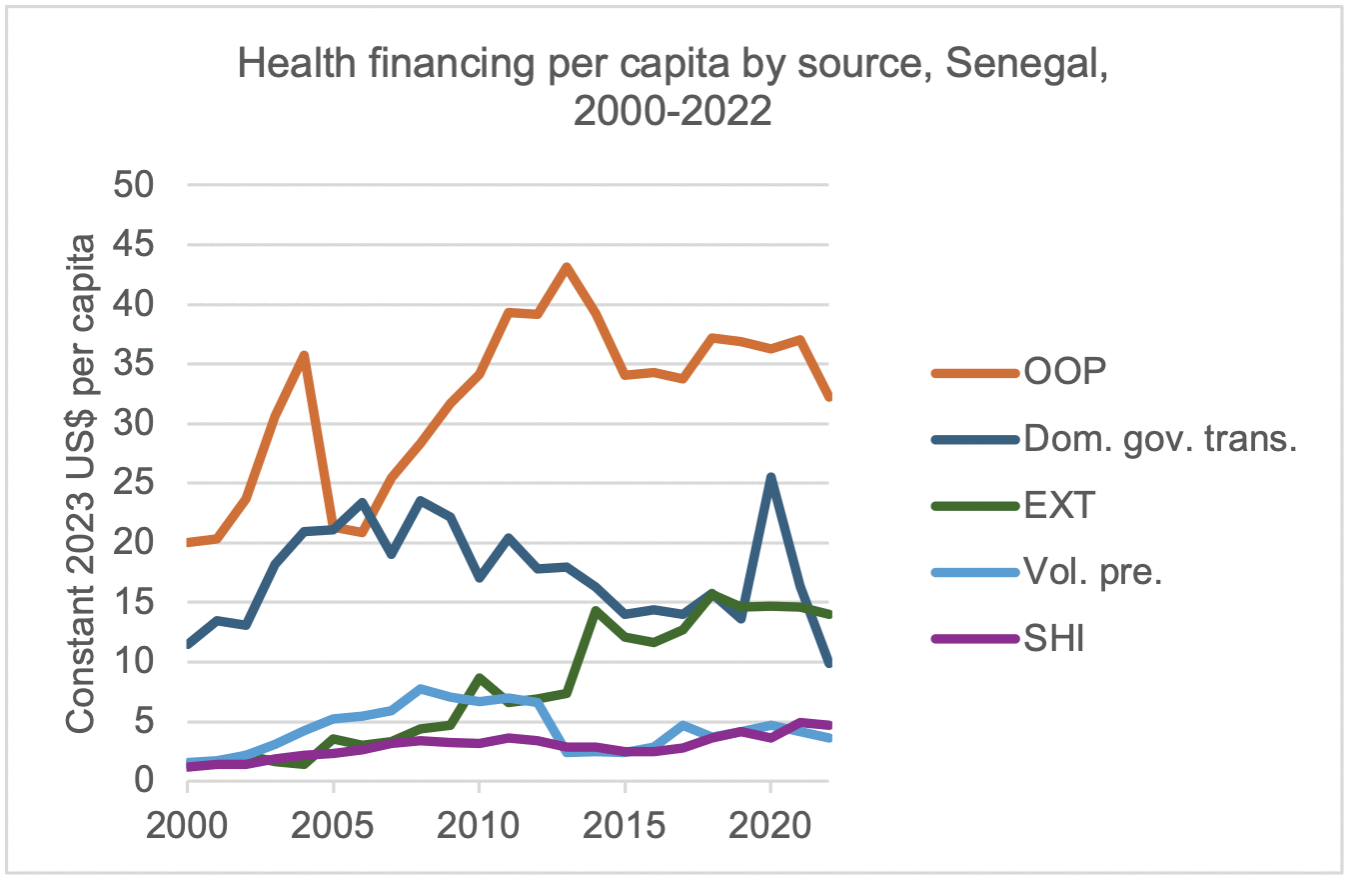
Health financing per capita by source in Senegal from 2000 to 2022 (constant 2023 US$) [8,14]. OOP: Out Of Pocket payments. Dom. gov. trans: Transfers from domestic government revenue allocated to health purposes. EXT: External health financing. Vol. pre: Voluntary prepayments. SHI: Social health insurance. Government subsidies to SHI are deducted from SHI figures. Data deflated using US$ GDP deflator data.

Lending to the economic and production sectors could have also indirectly supported increases in GHE-S by strengthening the foundation for raising revenue for the public budget, some of which could be spent on health. As development lending has increased (in itself a source of government revenue) (Fig 3), Senegalese government revenue has expanded from US$1.5 billion in 2000 to US$5.7 billion in 2023 (constant 2023 US$) (Fig 4) [49]. Senegalese GGE has grown from US$1.4 billion in 2000 to US$7.6 billion in 2022, while GDP has grown from $10 billion in 2000 to $31 billion in 2023 (constant 2023 US$) (Fig 2 and Fig 4) [8,49]. Against this economic expansion, GHE-S has however decreased substantially as a share of GGE, from 9.0% in 2000 to 3.4% in 2022 (Fig 4) [8,14]. In real terms, this translates into a decrease from $300 million in 2006 to $257 million in 2022 (constant 2023 US$), or from $26 per capita to $15 per capita (Fig 4 and Fig 5. *Note: Numbers deflated using US$ GDP deflator data for consistency across figures*) [8,14]. This real-term decrease in GHE-S does not lend support to the hypothesis of a positive indirect effect of lending to other sectors, although the counterfactual trend in the absence of lending is unknown.

### Has debt servicing constrained government health spending?

Our interview and document review findings point to a constraining effect of public debt servicing on government health spending. Government prioritisation of servicing its debt obligations was perceived by some interviewees as receiving higher priority than funding the health sector, along with making profitable investments to ensure economic growth. The education sector however appeared to suffer less from a debt servicing constraint than the health sector, possibly due to receiving higher government priority.

As seen in Fig 5, real-term OOP per capita expanded considerably in the late 2000s to remain the predominant source of health financing in Senegal. GHE-S has conversely decreased per capita since 2006 to US$15 in 2022 (constant 2023 US$), some of which has been picked up by development assistance, while Social Health Insurance (SHI) and Voluntary Health Insurance (VHI) remain at much lower levels (both less than $5 per capita in 2022 (constant 2023 US$)) [8,14].

For our observational time series data, no pattern was identified between year-on-year changes in PPG external debt servicing and total government current health spending (S1 Appendix) [8,14]. A negative relationship between debt servicing and government health spending was however identified by many interviewees (below). This indicates that the fiscal reality experienced by key informants may not easily be captured simply by observing year-to-year changes in debt servicing and health spending levels.

When comparing the two main social sectors, total government education expenditure has been consistently higher than total government health expenditure, and increasing in real terms over time (domestic and external source, including capital expenditure) (S1 Appendix) [8,14]. In 2021, total government education expenditure was 2-2.6 times total government health expenditure (S1 Appendix) [8,14]. ODA for health and for education have been at comparable levels since 2002 [50], and differences in ODA therefore do not explain the differences in government spending.

Across SSA, education sectors do receive more government funds than health sectors on average [8,14], however with a considerably lower ratio than in Senegal at about 1.3-1.4 on average (15.6% of GGE for education compared to 11.1-12.0% for health, 2021. *Note: Both education and health figures include domestic government current and capital spending, and external transfers via government. The lower bound for the ratio includes all external capital health spending as well, while the upper bound does not, as it is not specified whether these funds are distributed via government or not. To balance the risk of bias from measurement error versus sample representativeness, SSA countries with entire variables missing were removed for these calculations (n = 9), resulting in 40 remaining countries, some of which had individual values missing for some variables, in particular for capital health spending (n = 23)*) [8,14]. The Senegalese government thus appears to have given relative priority to the education sector, and any constraining effects of debt servicing present seem to still have allowed the government to provide significantly more and increasing levels of education spending compared to health spending.

An academic explained that the government prioritised the education sector above debt servicing and the health sector below both:

*“Health is* [number] *7 … in the hierarchy of priorities in Senegal … 1 is education, 2*^*nd*^ *is roads, the third is debt payment.”* (Academic)

This would help explain why the education budget would be less likely to be constrained by debt servicing requirements than the health sector as a result of government prioritisation.

Approximately half of participants, representing all stakeholder groups, believed that Senegal’s debt constrained the public budget, including the health budget. Some did not believe that this was the case, while others were not knowledgeable on the matter.

Several interviewees across all stakeholder groups believed a constraining effect of Senegal’s debt to impact across all sectors. A government official explained how the health sector was not exempt, and the government at times had to cut them to meet debt obligations.

*“We live it every day… Because we are obliged to pay the debt … we are obliged to make budgetary regulations on such sensitive sectors as health, education, even though we try to spare them.”* (Government)

A donor concurred:

*“Absolutely,* [debt obligations are] *going to affect the health sector because we know that these loans … the government of Senegal will have to respect their engagement in terms of timeline. If during this time … they have to pay back these loans, they don’t have enough resources, they have to cut somewhere. And when they cut, the health sector is also affected.”* (Donor)

The cited donor believed that IMF fiscal objectives compounded the effect of the debt on social sectors:

*“The IMF sets an objective to the government in term of what should be the level of debt and deficit each year. And so … the government needs to adjust its budget accordingly and so at the end of the day it’s the budget for social sectors that are cut.”* (Donor).

This interviewee did however attribute this mainly to the government and highlighted the change in attitude from the WB toward prioritising social sectors.

A constraining effect of debt on social spending in Senegal has been acknowledged in official country reports both when debt levels were highest around the turn of the millennium, and again in 2020. The 1998 Enhanced Structural Adjustment Facility policy framework paper recognised that “*the fiscal burden* [of external debt] *remains high, however, with debt service to revenues projected to be 38 percent in 1998. This makes it more difficult to increase expenditures, including for the priority sectors of education and health”* [68]. In the 2002 Poverty Reduction Strategy Paper (PRSP), it was similarly stated that: “*the country’s debt burden is a major obstacle to efficient allocation of public resources in favour of the social sectors*” [69]. In the recent context of Senegal’s growing debt burden, a 2020 IMF country report for Senegal stated that: *“… debt service continues to absorb a sizable portion of fiscal revenues, thus limiting room for other expenditures in critical areas such as infrastructure investment, health, and education”* [70].

#### Factors compounding debt servicing constraint of government health spending.

A few interviewees highlighted certain government prioritisations made under budget constraint, driving spending away from the health sector, some of which were thought to be exacerbated by debt servicing obligations. This included a focus on sectoral visibility, profitability, a belief in economic growth as first priority before social sectors, and cutting the health sector due to the presence of donors.

Firstly, an increased focus on sectoral visibility due to a debt servicing constraint on the government budget was explained by a CSO representative as politicians focusing spending where it would help secure re-election, when there was little left to spend:

*“If the money mobilized to repay the debt … returns to the donors, the government which does not have much means will prioritize … and they are not going to put it into health. They will put it in another much more visible sector to say that “I am building an airport, I am building roads”. … I have never seen a politician during an election campaign say that “I treated so many people” … They say: … “I don’t have a lot of money. The little I have, I put it in the sectors that allow me to be re-elected in the next elections”.* (CSO)

Secondly, lack of profitability of the health sector was also believed by the same interviewee to be an explanation for why the sector received less government priority and was subject to budget constraint from debt servicing obligations:

Q: *“Do you think the health sector is more affected than other sectors in terms of being constrained to pay off debt?”*

A: *“Yes I think so … As I said, health, the social, is not profitable. This is why we do not prioritize it.”* (CSO)

Another CSO leader concurred that health and social protection investments received last priority among government expenses, exacerbated by the weight of the debt. They described the conditions and priorities of the ministry of the budget as follows:

*From* [the small] *budgetary space you must first pay the debt, after that the security, the sovereign expenses, the operations, before being able to give water and health to the populations … The weight of the debt does not allow to invest in basic social services.”* (CSO).

Thirdly, a donor identified a dynamic in which the presence of donors to step in and cover costs in the health sector rendered this sector more likely to be subject to budget cuts during the financial year to meet debt obligations:

“[Cuts due to debt obligations] *can affect any sector, but specifically for the health sector, and the reason it’s going to affect more the health sector is the government knows that the health sector will receive more donor resources compared to the others”* (Donor).

## Discussion

With this study, we aimed to unpack how external official lending and debt have affected government health spending in Senegal. Our findings indicate that Senegal’s debt has constrained the general government expenditure budget and thereby the health sector budget. While development lending has supported the economic growth and government revenue and expenditure expansion seen since 2000, as well as directly supporting the health sector, any indirect economic effects have been largely cancelled out by decreasing relative budget prioritization of the health sector. Unexpectedly, the education sector appeared to have been insulated from debt effects due to a higher political prioritisation.

The majority of our study participants who held knowledge on the matter, across all stakeholder groups, believed that Senegal’s debt burden constrained the public budget, including the health budget. Official IMF and WB documents from 1998, 2002 and 2020 supported this assessment [68–70]. Given the consistent growth of Senegalese GGE, this finding should be interpreted as a relative consideration of the public expenditure growth rate, and the counterfactual of GGE in the absence of external official lending is not known. GHE-S however decreased as a share of GGE (from 9.0% in 2000 to 3.4% in 2022 (Fig 4) [8,14]), and it did not grow in real, absolute terms between 2006 and 2022 (using US$ GDP deflator [8]). This decoupling deviates from findings across 133 LMICs, showing that around 70% of the budgetary space for health is driven by changes in overall government expenditure, with the remaining 30% being attributable to the expenditure budget share allocation to the health sector [71]. In Senegal, the health sector’s share allocation has decreased so much that any effects from growth in GDP, revenue and expenditure have been largely cancelled out. Undoing this continuous regression on the Abuja pledge of 15% of the government budget being allocated to the health sector will be vital for making sustained progress on UHC and other health SDGs in Senegal.

Some of our interviewees helped explain these findings by saying that the government prioritised economic growth and servicing its external debt before funding the health sector. The health sector was also described as a sector with relatively low visibility and political value around elections. These political economy factors elicited via qualitative enquiry constitute “country specific features” that co-determine limited health budget prioritisation beyond statistical measures such as national income or perceived corruption [72]. In Ghana, where GHE-S is much higher per GGE and per capita, the performance of the National Health Insurance Scheme has been central to the fate of political parties around presidential elections [14,73]. In Nigeria however, where funding levels are comparable to Senegal, self-reinforcing beliefs of the superiority of the private health sector over an underfunded public health sector have been pervasive [14,74]. Further qualitative research building on studies such as [73,74] that directly seek to identify the most effective levers and arguments for strengthening political commitment to health sectors in Senegal and other parts of the ECOWAS region are needed to help promote progress on the Abuja pledge [43].

If the cycle continues of lending primarily for economic growth and meeting increasing debt obligations before investing in the health sector, the outlook for a revitalisation of the budget priority given to the health sector in Senegal appears limited. While investments in the highly prioritised education sector could help support economic growth and thereby the revenue basis for GHE-S, such synergies were not reflected in trends in GHE-S over time in the period of study. They could however materialise in the future, provided GHE-S receives increasing budget priority out of GGE.

Our finding of debt constraint of the health budget extends some previous findings by other authors. Two studies of the period 1970–2000 found that the Senegalese government spent around 20 percent of its resources on debt servicing [75,76], and that a one percent increase in debt servicing was associated with a reduction in total domestic expenditure by 0.13 percent of GDP [75,76]. With growing concerns of a Senegalese debt crisis, the identified past effects could have become worse over time [44,77]. Our findings also align well with existing case studies from Kenya [33,34], and with multi-country econometric studies, generally finding negative associations between debt variables and government health spending [2,4,26–32] (one study has had more variable findings [3]). By taking a mixed methods approach with new primary data generation in a new country context through key informant interviews, combined with in-depth secondary data exploration, our study was able to expand on the existing literature by identifying and unpacking the fiscal implications on health spending from external lending at the country level. Beyond the identified debt constraint, our study also provided empirical evidence for the expected though essential point that loans may benefit the health sector in the first place. This is important for a more nuanced understanding of the full life-cycle impacts of development lending on health financing. The more the portfolio of government development loans are directed toward the health sector in a sustained manner over time, the less likely they are to cause a net erosion of the government health budget when loan repayments are taken into account (adjusted for inflation and value discounting). This is moderated by the revenue-expanding effects of development lending for economic growth, provided the added revenue benefits the health sector, which we did not find evidence for in Senegal in the period of study.

Importantly, by showing how government education spending kept on growing in spite of debt obligations as opposed to health spending, our study provided an original empirical basis for the interpretation that a constraining effect of debt on spending in a given social sector may be modified or even fully negated by political priority of that sector. This is a novel finding which leads to policy recommendations as seen in the conclusions section. It deviates from most of the reviewed econometric literature on public debt and education spending, mostly finding a negative link [26,27,35–41]. This illustrates the importance of an appreciation of the political economy and fiscal prioritisations made at the individual country level, and the challenges of extrapolating findings from the cross-country level or from other individual countries.

Additionally, it deserves mention that while this paper focused on the modality of development lending, we acknowledge the important EDP contribution of providing grant financing. In spite of its decreasing share over time, it has provided 81% of ODA for the health sector in Senegal on average between 2002–2023 [50]. The significant overall ODA funding flows to the health sector in Senegal offer another explanation for why this sector has not been prioritised by the Senegalese government through aid displacement of government funds (fungibility, discussed for Senegal in another article [78]) [31,32,78–91]. This has the same negative direction of effect on health financing as the identified debt constraint, constituting a dual challenge for revitalising health spending in Senegal, and potentially in other countries experiencing similar fiscal conditions. Importantly, a donor pointed to a possible point of negative synergy, whereby they deemed the health budget more likely to be cut in order to meet debt obligations because of the presence of donors to cover costs. While not brought up by other interviewees, this observation warrants further exploration both in Senegal and elsewhere, as a potentially detrimental interaction between these two external macroeconomic factors.

Finally, the recent cuts to ODA from the United States, France, Germany and other donors will leave a funding shortfall across sectors in Senegal [92,93]. To mitigate this and avoid adverse consequences for the Senegalese people, other partners and/or the Senegalese government will have to step in and cover costs. The latter adds the risk of necessitating external borrowing, which could further fuel downstream debt constraints on the health sector, in turn strengthening the case for debt relief in Senegal (see Conclusions section).

### Limitations

Unpacking our macroeconomic and fiscal dynamics of interest is inherently complicated, with complex causal chains, numerous contemporaneous determinants and competing political considerations. This served as the motivation for our multipronged methodological approach and led us to focus more on identifying and discussing different pathways of effect while remaining cautious about drawing causal inferences.

Using descriptive statistics based on relatively short observational time series to quantitatively assess sectoral impacts from cross-sector fiscal level burdens proved challenging. This is an inherent statistical limitation when using single-country observational macroeconomic data, further complicated by the fact that debt impacts the general public budget before sectoral allocations. Using complementary data sources such as individual stakeholder accounts or written reports were necessary to discuss possible causal relationships.

We explored the feasibility of a formal econometric analysis, however, the short time series with limited degrees of freedom rendered a theoretically justified multiple regression model infeasible due to limited power and risk of overfitting, while a more parsimonious specification risked omitted variable bias and associated erroneous statistical inference.

Importantly, a series of audits have revealed that Senegal’s public debt had been underreported by the previous administration, adjusting debt-to-GDP estimates up to a critical 119% by end-2024 [94–96]. In light hereof, our quantitative findings for Senegal’s debt burden may serve as a lower bound, its impacts on government health spending may be even larger than our findings suggest, and these may become worse over time unless the debt burden is addressed.

In interviews, five participants did not consent to recording of the interview or quoting them, so instead FF took notes and used these interviews to broadly further his understanding of the research questions and the interviewee’s position in regard hereto. Also, potential interviewees in one government agency, two hospitals, two multilateral organisations and two academics did not respond or were unavailable. However, our interview group for this study was overall well balanced, with 8 development partners, 9 government representatives, and 8 non-government representatives.

## Conclusions and recommendations

Our findings indicate that Senegal’s debt has constrained the general government expenditure budget and thereby the health sector budget. While development lending has supported economic growth and government revenue and expenditure expansion, as well as directly supporting the health sector, any indirect economic effects have been largely cancelled out by decreasing relative budget prioritization of the health sector. Unexpectedly, the education sector appeared to have been insulated from debt effects due to a higher political prioritisation.

This research has added to a limited set of case studies of the impacts of debt on government health spending in SSA. We were able to significantly expand on the existing literature in particular in eliciting key stakeholder accounts of the fiscal reality in Senegal, and providing a full view of influence from initial lending to debt servicing obligations, the first of which is often neglected in the literature. The identified relative insulation of the education sector from debt servicing obligations due to political priority illustrates how quantitative studies grouping all social sector spending as a single outcome variable may miss important sectoral effects.

While development lending has directly benefited the health sector in Senegal, the health sector should be protected from unprecedented public external debt servicing levels. This responsibility falls both on domestic policy makers, EDPs and the IMF. Enough flexibility should be made in repayment timelines to avoid the government having to consider cutting social sectors in order to make repayments. Exploring options for debt relief to address the rapidly growing debt burden of Senegal may become necessary, however our findings indicate that lower debt servicing levels do not equate increased health spending without political priority. Mechanisms such as debt-to-health swaps could be considered to ensure the health sector would benefit from any funds made available through debt relief [23]. This could also help level out the persistent and growing imbalance between the health and education sectors seen and prevent the health sector from losing out behind what is perceived as more economically profitable sectors.

As long as economic growth and servicing its now historical debt levels are top priorities for the Senegalese government to meet its obligations to EDPs, the IMF and private creditors before investing in the health sector, substantive real-term GHE-S gains and expansion of public-sector driven UHC expansion appear unlikely. Supported by debt relief, increased government priority of the health sector will be necessary to break this cycle, working toward the Abuja pledge of 15% of the government budget being allocated to the health sector [43] - ideally from domestic revenue. This kind of fiscal policy turnaround may be a necessary precondition for sustained progress on UHC in Senegal. In advocacy work and discussions with the Senegalese government, using the substantial literature that establishes health as a key driver of economic productivity might be helpful for making the case for increased health investments (e.g. [97]).

Additionally, recent ODA cuts from several donors may necessitate further external borrowing to cover costs. This strengthens the case for A: relief of existing debt obligations, and B: increasing the availability of concessional grant and loan financing from other partners, in order to avert unsustainable debt levels and exacerbated constraint on the health sector.

Future research avenues include exploring the lending and debt implications for the health sector in other countries using qualitative and mixed methods to understand individual country realities. Such case studies could focus on countries at high risk of or in debt distress, also exploring how implications of debt translate at different levels of the health system. Countries may respond and prioritise differently when burdened by debt, and understanding individual country impacts may be important for informing both creditor policy to avoid averse social impacts, and to inform a redistribution of any freed-up funds in future debt resolution processes toward sectors and programs that have been heavily impacted by debt.

Importantly though, keeping in mind the range of beneficial effects of concessional development lending, which had also benefited the health sector in Senegal, is essential for a nuanced and complete understanding of the health financing implications of development lending.

In Senegal, the determinants underlying the relative prioritisation of the education sector over the health sector and apparent differential insulation from debt impacts warrants further exploration. This would likely benefit from applying a broad political economy lens encompassing both domestic and external drivers.

## Supporting information

S1 Appendix Supplementary methods, results, and interview materials. (DOCX)

S1 Checklist Inclusivity in global research questionnaire. (DOCX)
