# Supplementary material for: Influence of public external debt on government health expenditure: A mixed-methods case study of Senegal
Source: PLOS Glob Public Health. 2026 Jul 10;6(7):e0006698. doi: 10.1371/journal.pgph.0006698 (PMC13353989; doi:10.1371/journal.pgph.0006698)
Supplement: S1 Appendix — (DOCX) [file pgph.0006698.s001.docx]

## Appendix: Supplementary methods, results, and interview materials

#### Health system organisation in Senegal

As elsewhere, the Senegalese health system is organised in a pyramidal form, with a primary (peripheral), secondary (intermediary) and tertiary (central) level, both in terms of management and service provision (Figure A1) [1].

**Health management & administration**

Ministry of health, secretariats, directorates and national services

Medical regions, regional services

Health districts, departments and centres

**Health service provision**

Level 3 public health institutions

*- Tertiary referral hospitals*

Level 2 public health institutions

*- Regional hospitals*

Level 1 public health institutions

*- Health centres*

*- Health posts and clinics*

Central

(tertiary)

Intermediary

(secondary)

Peripheral

(primary)

Figure A1: Health system pyramid in Senegal. Adapted from “Situation Economique et Sociale du Senegal 2019”, Agence National de la Statistique et de la Demographie (2022) [1]. Licensed under CC BY 4 (https://creativecommons.org/licenses/by/4.0/). Changes made from original figure.

## Search strings

(international OR external OR official OR development) AND (loan* OR credit* OR lend* OR borrow* OR debt OR conditionalit*) AND Senegal.

*Carried out in March 2023, repeated in August 2023, and on March 4 2025*

(Loan* OR credit* OR lend* OR borrow* OR debt) AND Senegal

*Run on March 4 2025*

(Loan* OR credit* OR lend* OR borrow* OR debt) AND health AND (financ* OR expenditure OR spend*) AND Senegal

*Run on March 4 2025*

## Notes for Figure 2

The below caption provides additional details for the numbers underlying Figure 2 (shortened to meet journal formatting requirements).

Figure 2: **Panel A**: Public and Publicly Guaranteed (PPG) borrowing in Senegal separated by creditor type, 1970-2023 (billion constant 2023 US$) [3, 7]. Data are for disbursements, i.e. transfers made in each year (flows, as opposed to stock in Panel B). IMF loan disbursement data is only available for drawings made on the IMF general resource account, not the reserve tranche. **Panel B**: PPG debt stock in Senegal from 1970-2023 [7]. IMF credit includes all IMF loans except amounts drawn against the country’s reserve tranche position. It includes Special Drawing Rights (SDR) allocations [7]. Note: There may be some overlap between “Bonds” and the other creditor categories as these may buy Senegalese bonds, however we were unable to identify more detailed data on the owners of Senegalese bonds to be able to avoid double counting. **Panel C**: Gross Domestic Product (GDP) (1970-2023), PPG external debt stock (1970-2023), and General Government Expenditure (GGE) in Senegal (1994-2022) [3, 49]. **Panel D**: PPG external debt service (1970-2023) and Government Health Expenditure as a Source (GHE-S) (2000-2022) in Senegal [3, 9]. (Data deflated using WB US$ GDP deflator [3]. Note: PPG external debt does not include the use of IMF credit [7]).

## Participant information sheet

[INSTITUTIONAL DETAILS REMOVED]

***PARTICIPANT INFORMATION SHEET***

**Study title: *Official Development Assistance, External Public Debt and Equity in Domestic Health Financing in Low- and Middle-Income Countries: Panel Data Analysis and Case Study in Senegal***

**Primary funder:** [Funder details]

**Principal Investigator (PI) :** [Author details]

**Co-PI:** [Author details]

**Background and aims**

The composition of domestic financing for health in Senegal is shaped by a broader political and economic context. This research aims to study the role of international donors and creditors in determining this financing in Senegal. We intend to publish the results of this research.

**Methods**

This case study of Senegal consists of interviews with representatives and document reviews at ministries and health policy institutions at the regional and district level, international official donor and creditor institutions, academics and health professionals, and civil society organizations in Senegal.

**Participation**

You have been invited to participate in this study because we believe you may be knowledgeable about some of the dynamics between international donors and creditors and the Senegalese health sector. By participating and sharing your knowledge, you will be able to contribute to our understanding of this topic. By participating in this study, you will not be putting yourself or anyone else at any risk. Participation is completely voluntary. If you agree to participate, you may still withdraw at any time without giving a reason. The interviewer will take notes during the interview. We would like to record the interview and transcribe the interview for our analysis, however, you may ask for the interview not to be recorded. In that case, the interviewer will only take hand-written notes without recording during the interview.

**Confidentiality**

You have the option of being anonymous or to have your identity reported in the publications we will write from the research. If you choose to be anonymous, you may choose from different levels of anonymity: You can choose to only have your name anonymized, to also have your position in the organization you work in anonymized, and to have the organization you work in anonymized as well. You can further ask that we don’t quote you at all, not even anonymously, and in that case we will only use the information you provide us to inform our analyses but with no direct citation or reference. [Author] (the principal investigator) and a transcriptionist will be the only persons who will have access to the recordings produced from the interview. The transcriptionist will be bound by a confidentiality agreement that will make her/him unable to disclose any information about you to third parties. All raw interview recordings will be destroyed at the end of the project.

Please consider whether you would like to remain anonymous, and if so the degree of anonymity you would prefer.

You will also have the opportunity to decide whether the transcripts themselves, i.e. your words on paper, can be made publicly available in anonymized form in our secure online data repository at the end of the project. If you accept this, we will anonymize the transcript according to your choice in the participant consent form.

Any questions you may have will be answered during the interview. If you have any questions in the meantime, please feel free to contact [Author] using the information below.

**Further information**

If you have any questions, please contact:

[Author details]

## Ethical consent form

## [INSTITUTIONAL DETAILS REMOVED]

***ETHICAL CONSENT FORM***

**Study title: *Official Development Assistance, External Public Debt and Equity in Domestic Health Financing in Low- and Middle-Income Countries: Panel Data Analysis and Case Study in Senegal***

**Primary funder:** [Funder details]

**Principal Investigator (PI):** [Author details]

**Co-PI:** [Author details]

**Participant identification number**: |__|__|__|__|__|__|__|__|__|__|__|__|

(Participant name)

1. a.  I have read the information sheet for this study and understood the information provided

**OR**

1. b.  The study personnel have informed me in a language I understand

**AND I**

- confirm that my choice to participate is entirely voluntary,
- understand that I may withdraw from this study at any time without giving a reason,
- confirm that I have had the opportunity to ask questions about this study and that I am satisfied with the answers provided,
- understand that I authorize the persons described in the information sheet to access the interview recording and transcript (in the case that I authorize recording of the interview),
- have had time to think about whether I would like to participate in this study,
- agree to participate in this study.

1. I agree that this interview can be recorded.

3. Please read the following options carefully and tick **ONE**:

a.  I agree that the contents of this interview can be quoted and that these quotations can be attributed to me

b.  I agree that the contents of this interview can be quoted, but I would like my name to be anonymized, although you can mention my organization and my position within it

c.  I agree that the contents of this interview can be quoted, but I would like my name and my position within my organization to be anonymized, although you can mention my organization

d.  I agree that the contents of this interview can be quoted, but I would like my name, position and organization to be anonymized. However, you may refer to me as "a representative of Ministry X", "a representative of a donor agency", "a representative of a creditor agency" or "a representative of an NGO", as the case may be

(if other, please indicate: ……………………………………………………………………)

 e.  I agree that the contents of this interview can be quoted, but I would like my name to be anonymized, as well as any information that can be used to identify me, including my organization and my position within it

 f.  I do not agree that the contents of my interview can be quoted, but the researchers can use the information from my interview to inform their analyses

4. Do you allow the transcript of your interview to be made available in a secure online data repository? If so, we will follow the level of anonymity you have selected above, for example, if you have selected option "c" above, we will delete any information that allows others to identify your name and your position within your organization.

a.  Yes
 b.  No

Full name: ………………………………………………………….…
Date (dd/mm/yy): ………..……………… Time (24h) :………………….
Signature: …………..…………………………………………………….

To be completed by the person obtaining the consent:

Name of person obtaining consent: ............................................................................................

I confirm that I have explained the study information accurately in .................................................................. and that the participant has understood it to the best of my knowledge.

He/she has freely consented to participate.

*Name:* ……………………...…………………………………………….…

*Date (dd/mm/yy):* ………..……………… *Time (24h) :*………………….

*Signature:* …………..…………………………………………………….

## Example interview topic guide

**Interview guide for donors in Senegal**

**Study title: *Official Development Assistance, External Public Debt and Equity in Domestic Health Financing in Low- and Middle-Income Countries: Panel Data Analysis and Case Study in Senegal***

**General information**

Country of origin:

Place of residence:

1. Icebreaker
   1. What is your role in this institution?
   2. How long have you been working here?
   3. Have you held any other positions here? (Which ones?)
2. Health sector financing
   1. What are the priority areas of [NAME OF ORGANISATION] for your support to the health sector in Senegal? (Why do you give priority to these areas?)
   2. In regard to the topic of health financing, or more broadly health economics, does [NAME OF ORGANISATION] have any specific projects/ programs in this area in Senegal? (Can you tell me about this?)
   3. What about universal health coverage and access to health services? Do you have any programs in this area? (Can you tell me about this?)
   4. Why has [NAME OF ORGANISATION] chosen to support the programs you just mentioned? (What are their qualities of these programs) ?
   5. How does [NAME OF ORGANISATION] want the balance between public and private health financing in Senegal to develop over time: Towards higher dependence on private or public funding? Why?
   6. Who does [NAME OF ORGANISATION] think should contribute the majority of health funding in Senegal? (Government, donors, patients)? Why ?
3. Involvement of international stakeholders in determining the public health budget
   1. In your opinion, does the [NAME OF ORGANISATION] have a say when the overall size and distribution between the different expenditure items are decided for the health budget in Senegal? (Please explain)
   2. In your opinion, which stakeholders outside of the government have the most influence on these decisions? (Donors, creditors, civil society, voters)? (Why do you think so)?
   3. Under your impression, what are their main priorities or objectives in these processes?
   4. Do you see any conflicting priorities between these international actors’ and the government’s priorities with regard to Senegal's health budget? (Which ones?)
   5. Do you see any mutually contradictory priorities between the different international actors, including yours, with regard to the health budget? (Which ones?)
4. Fungibility
   1. Does the [NAME OF ORGANISATION] have an opinion on how national resources for a sector should be spent when development assistance for that sector is received, i.e. should the government increase its funding, keep at same level, or lower it? Can you tell me about this?
   2. Have you ever seen a decrease in domestic government funding for the health sector after having received development assistance for this sector? Can you tell me about this ?
      1. *If yes:* How has this affected the health sector?
      2. *If yes*: What happens when the aid is exhausted? Do you normally see domestic funding increased back to the initial level so that health services can continue?
      3. *If yes*: Do you think that this reduction in domestic funding, when development assistance is received, affects access to health services? If so, how? (For the poor, in rural areas?)
   3. *(If applicable): The effects you mention, when have they mainly occurred?*
      1. Are they still happening today?
   4. In your experience, does the [NAME OF ORGANISATION] have an influence on how the Senegalese government spends its own money when you provide development assistance for a sector? In what ways?
   5. *(If applicable) : What is your opinion on this redistribution of funds? Why ?*
5. Loans and debt repayments
   1. In your experience, which sectors have development loans received from international financial institutions (International Monetary Fund (IMF), World Bank, African Development Bank) and bilateral creditors mainly benefited?
   2. In your experience, have the development loans received from these agents benefited the health sector? (In what way?)
   3. Do you think that the debt owed to external official creditors and the repayments made on this debt affect the Senegalese health budget?
      1. *If yes*: In what ways?
      2. *If yes*: Do you think it affects the health sector more than other sectors? Why/ why not?
      3. *If yes*: And how do you think this may affect access to health services? ( For the poor, in rural areas?)
      4. *If yes*: Do you think this is a growing, stable or diminishing problem? Why?
6. IMF / World Bank Programs and loan conditionalities
7. Today, Senegal is part of IMF and World Bank programmes and is subject to the policy conditionalities necessary to be able to lend from these institutions. Do you think that these programmes with their conditionalities have affected Senegal's health sector?
   1. If yes: In what ways?
   2. If yes: Do you think these conditionalities have affected access to health services? (In what ways?) (For the poor, in rural areas?)
8. Are you aware of any conditionalities imposed by IMF/WB programs directly on the health sector?
   1. If yes: How do you think these conditionalities have affected the health sector?
   2. If yes: Do you think these conditionalities have affected access to health services? (How?) ( For the poor, in rural areas?)
9. Some countries have experienced national wage ceilings [*explain*] for the health sector as part of these programs. Do you know if this has been the case for Senegal? (Can you tell me about them?)
10. What about the privatization of health facilities due to loan conditionalities from the IMF/WB? (Can you tell me about this?)
11. What about the introduction of user fees for health services? (Can you tell me about this ?)
    1. (*If applicable*): How do you think these things have affected access to health services in Senegal? (For the poor, in rural areas?)
    2. (*If applicable*): The effects you mention, when have they mainly occurred?
       1. Are they still happening today?
12. Last question
    1. Is there anyone else you would recommend I interview ?

**Thank you**

#### Supplementary results

Figure A2: Share of official development assistance provided as loans in Senegal from 2002-2023 [2]. “Health” includes both the categories “Health” and “Population policies/Programmes & reproductive health”.

**B**

**A**

Figure A3: Panel A: Selected government sectoral spending and Public and Publicly Guaranteed (PPG) external debt service in Senegal, 2000-2022 (constant 2022 US$) [3, 4]. Panel B: Year-on-year changes in government spending for same sectors and PPG external debt service in Senegal, 2001-2023 [3, 4]. Figures include external transfers via government. Education and military expenditure also include government capital expenditure, which is not available for the full time series for the health sector, so figure only shows total government current account health expenditure (domestic and external source). For 2013-2021, total capital health expenditure via the government was at least US$19-90 million (lower bound, excluding external) and maximum US$46-239 million (upper bound, including all external, some of which may have been channelled outside of the government) (constant 2023 US$). In 2021 specifically, domestic government capital health expenditure was US$37 million, and external capital health expenditure was $202 million, some of which may have been channelled outside of the government (constant 2023 US$).

References

1. Agence nationale de la statistique et de la demographie (ANSD). Situation economique et sociale du Senegal 2019. 2022 [cited 2022 January 20]. Available from: <https://www.ansd.sn/sites/default/files/2022-04/4-SES-2019_Sante.pdf>.

2. OECD. Creditor Reporting System (CRS). 2024 [cited 2023 February 16]. Available from: <https://stats.oecd.org/Index.aspx?DataSetCode=CRS1>.

3. World Bank. World Bank Open Data. 2025 [cited 2025 July 25]. Available from: <https://data.worldbank.org/>.

4. WHO. Global Health Expenditure Database. 2024 [cited 2024 March 28]. Available from: <http://apps.who.int/nha/database>.
