# Supplementary material for: Influence of public external debt on government health expenditure: A mixed-methods case study of Senegal
Source: PLOS Glob Public Health. 2026 Jul 10;6(7):e0006698. doi: 10.1371/journal.pgph.0006698 (PMC13353989; doi:10.1371/journal.pgph.0006698)
Supplement: Attachment — Submitted filename: 5PGPH-D-25-03785.pdf [file pgph.0006698.s004.pdf]

PLOS Global Public Health

Influence of Public External Debt on Government Health Spending: A Mixed-Methods Case Study of Senegal

--Manuscript Draft--

**Manuscript Number:**	PGPH-D-25-03785
**Article Type:**	Research Article
**Full Title:**	Influence of Public External Debt on Government Health Spending: A Mixed-Methods Case Study of Senegal
**Short Title:**	Debt and Health Spending in Senegal
**Corresponding Author:**	Frederik Federspiel, MD, MPH, PhD<br>London School of Hygiene & Tropical Medicine Faculty of Public Health and Policy<br>London, UNITED KINGDOM OF GREAT BRITAIN AND NORTHERN IRELAND
**Order of Authors:**	Frederik Federspiel, MD, MPH, PhD
	Josephine Borghi, MSc, PhD
	Henning Tarp Jensen, MSc, PhD
	Elhadji Mamadou Mbaye, PhD
	Melisa Martinez-Alvarez, MSc, PhD
**Keywords:**	Debt; health spending; Senegal; mixed-methods
**Abstract:**	Background<br><br>Public external debt burdens of low- and middle-income countries have grown substantially over the past decade linked to increased borrowing. Our empirical understanding of how external official loans and their ensuing debt burden may impact government health spending at the individual country level is limited. We conducted a mixed-methods case study of Senegal to better understand these relationships.<br><br>Methods<br><br>We performed descriptive quantitative analyses of loan, debt and health financing statistics in Senegal from 1970-2023. We also conducted semi-structured interviews between October 2019 and January 2020 with 25 key informants from government ministries, development partners, academia, civil society and hospital management in Senegal, and a purposive document review of reports from government websites and academic literature.<br><br>Results<br><br>Debt obligations resulting from external loans appear to have had a constraining effect on the government budget including for health. Development loans have however also supported the health sector directly. We found less support for an indirect benefit to the health budget through loans to other sectors, due to substantial decreases in government health expenditure out of general government expenditure from 2000 to 2022.<br><br>Conclusions<br><br>To mitigate the negative effect of debt repayment, official external creditors should consider more flexible debt repayment timelines and exploring options for debt relief including debt-to-health swaps. Supported by debt relief, increased government priority of the health sector working toward the Abuja pledge will be necessary to make progress on universal health coverage. We encourage further country level research documenting debt impacts on health financing.
**Additional Information:**	

Powered by Editorial Manager® and ProduXion Manager® from Aries Systems Corporation

Question	Response
**Financial Disclosure**<br><br>Enter a financial disclosure statement that describes the sources of funding for the work included in this submission and the role the funder(s) played. This includes grants and any commercial funding of the work or authors.<br><br>This statement will be typeset if the manuscript is accepted for publication.<br><br>*Please review the submission guidelines and the instructions link below for detailed requirements and guidance.*	This research was funded by The Economic and Social Research Council (ESRC) Doctoral Training Partnership (DTP) (FF), Reinholdt W. Jorck og Hustrus Fond (FF), Torben og Alice Frimodts Fond (FF), Christian Larsen og Dommer Ellen Larsens Legat (FF), The Anglo-Danish Society Scholarship (FF), Den Raben-Levetzauske Fond (FF), Christian og Ottilia Brorsons Rejselegat for yngre videnskabsmænd- og kvinder (FF), and Carl og Ellen Hertz' legat til Dansk Læge- og Naturvidenskab (FF).<br>The funders had no role in study design, data collection and analysis, decision to publish, or preparation of the manuscript.
**Competing Interests**<br><br>On behalf of all authors, disclose any competing interests that could be perceived to bias this work.<br><br>This statement will be typeset if the manuscript is accepted for publication.<br><br>*Please review the instructions link below and PLOS Global Public Health's competing interests policy to determine what information must be disclosed at submission.*	The authors have declared that no competing interests exist.
**Data Availability**<br><br>Before publication, Authors are required to make fully available and without restriction all data underlying their findings. Please see our PLOS Data Policy page for detailed information on this policy.<br><br>A **Data Availability Statement**, detailing where the data can be accessed, is	The participant information sheet, ethical consent form and example interview topic guide used for this study are made available at the LSHTM Data Compass [1] and in the appendix.<br>The data used for this article can be shared in anonymous form on reasonable request to the corresponding author.<br>The participant information sheet, ethical consent form and example interview topic guide used for this study are made available at the LSHTM Data Compass [1] and in the appendix.<br>The data used for this article can be shared in anonymous form on reasonable request to the corresponding author.<br><br>1.London School of Hygiene and Tropical Medicine. LSHTM Data Compass. 2025 [cited 2025 August 1]. Available from: https://datacompass.lshtm.ac.uk/id/eprint/3698/.

required at first submission. Insert your Data Availability Statement in the box below.<br><br>Please see the data reporting section of our submission guidelines for instructions on what you need to include in your Data Availability Statement.<br><br>This statement will be typeset if the manuscript is accepted for publication.<br><br>*PLOS allows rare exemptions to address legal and ethical concerns. If you have legal or ethical restrictions, please use the box below to detail these in full sentences for the Journal team to consider.*	

Manuscript

Click here to access/download;Manuscript;Senegal_debt_healthspending_P

Title: Influence of Public External Debt on Government Health Spending: A Mixed-Methods Case Study of Senegal

Short title: Debt and Health Spending in Senegal

Authors:

Frederik Federspiel[a], MD, MPH, PhD (corresponding author)

lsh1601722@alumni.lshtm.ac.uk

Josephine Borghi[a, b], MSc, PhD Josephine.Borghi@lshtm.ac.uk

Henning Tarp Jensen[a, c], MSc, PhD Henning.Tarp-Jensen@lshtm.ac.uk

Elhadji Mamadou Mbaye[d, e], PhD elhadjimamadou.mbaye@iressef.org

Melisa Martinez Alvarez[a, f], MSc, PhD Melisa.Martinez-Alvarez@lshtm.ac.uk

a: Dept. of Global Health and Development, London School of Hygiene and Tropical Medicine, 15-17 Tavistock Place, London, WC1H 9SH, United Kingdom

b: International Institute for Applied Systems Analysis, Schlossplatz 1, Laxenburg A-2361, Austria

c: Department of Food and Resource Economics, University of Copenhagen, Rolighedsvej 23, Frederiksberg 1958, Denmark

d: Department of Political Science, Gaston Berger University, Route de Ngallèle, Saint Louis BP 234, Senegal

e: Institut de Recherche en Santé, de Surveillance Epidemiologique et de Formation (IRESSEF), Arrondissement 4 Rue 2 D1, Pole Urbain de Diamniadio, Dakar BP 7325, Senegal

f: Independent consultant.

Author contributions

Conceptualization – FF, JB, HTJ, EMM, MMA

Data curation – FF, EMM, MMA

Formal analysis – FF

Funding acquisition – FF

Investigation – FF

Methodology – FF, JB, MMA,

Project administration – FF, MMA

Resources – FF, EMM, MMA

Software - FF

Supervision – JB, HTJ, MMA

Validation – FF

Visualization - FF

Writing - original draft - FF

Writing - review & editing – FF, JB, HTJ, EMM, MMA

Abstract

Background

Public external debt burdens of low- and middle-income countries have grown substantially over the past decade linked to increased borrowing. Our empirical understanding of how external official loans and their ensuing debt burden may impact government health spending at the individual country level is limited. We conducted a mixed-methods case study of Senegal to better understand these relationships.

Methods

We performed descriptive quantitative analyses of loan, debt and health financing statistics in Senegal from 1970-2023. We also conducted semi-structured interviews between October 2019 and January 2020 with 25 key informants from government ministries, development partners, academia, civil society and hospital management in Senegal, and a purposive document review of reports from government websites and academic literature.

Results

Debt obligations resulting from external loans appear to have had a constraining effect on the government budget including for health. Development loans have however also supported the health sector directly. We found less support for an indirect benefit to the health budget through loans to other sectors, due to substantial decreases in government health expenditure out of general government expenditure from 2000 to 2022.

Conclusions

To mitigate the negative effect of debt repayment, official external creditors should consider more flexible debt repayment timelines and exploring options for debt relief including debt-to-health swaps. Supported by debt relief, increased government priority of the health sector working toward the Abuja pledge will be necessary to make progress on universal health coverage. We encourage further country level research documenting debt impacts on health financing.

Keywords

Debt, health spending, Senegal, mixed-methods

Introduction

In the late 1990's and early 2000's, the public external debt burdens of Low- and Middle-Income Countries (LMICs) were high. This was viewed as unsustainable, constricting fiscal space in turn limiting investments in social sectors. Debt relief was granted to help countries free up resources to develop and fund their hard-pressed education and health sectors [1, 2]. This resulted in falling debt obligations during the 2000s [3-6]. The debt burdens of LMICs to foreign creditors have since been growing over the following decade, as debt relief decreased and public external borrowing increased [7]. In 2022, 49 LMICs spent more on public external debt servicing than on health compared to 33 in 2012 (out of 112 LMICs with data available for both years) [8, 9]. Across LMICs, real public debt interest payments per capita surged during the COVID-19 pandemic, with further increases projected up until 2029 especially in Upper-Middle-Income Countries (UMICs) [10]. Recent calls have been made for debt resolution, including by the United Nations and the WB, to ensure funding can be sustained for public sectors, including health sectors (e.g. [11-18]).

Existing LMIC literature has examined the association between public debt and government health spending through quantitative cross-country panel data analyses in African [19-22], Latin American [23], South- and Southeast Asian [24], and select "emerging" and middle-income countries [25] as well as across LMICs overall [26-28]. Mixed-methods and quantitative reports have also examined the debt burden and government health spending in Kenya [29, 30]. The majority of these studies find negative associations between debt burdens and government health spending. This is similar to most of the identified econometric literature on public debt and education spending, mostly finding a negative link [19, 20, 31-37].

The existing quantitative, cross-sectional, multi-country[1] analyses are however limited in their ability to assess individual country budget realities, prioritisations made and the underlying reasons for this. As seen, there are limited country case studies of the impacts of debt obligations on government health spending in LMICs. With global debt burdens growing, understanding individual country realities as they balance financial obligations to their external creditors and to their health sectors is increasingly important. How do governments prioritise, are they able to protect the health sector from debt obligations and if so how, and is the health sector equally impacted as for example the education sector? What policy lessons can be learnt from these individual country experiences? Sub-Saharan Africa (SSA) in particular is vulnerable with relatively low levels of health spending, growing debt burdens, and a relatively low resilience to economic shocks [8, 9]. On an indebted continent, our case country of Senegal is among the most indebted [3], allowing for a highly topical exploration of these

[1] [32] and [35] are single-country studies.

questions under conditions of a high debt burden, with potential lessons for other countries on a similar track.

Our study aims to understand the influence of external official loans and the ensuing debt obligations on domestic government health spending (Government Health Spending as a Source, GHE-S) in the context of an indebted SSA country, by performing an in-depth mixed-methods case study of Senegal [9, 38].

Materials and Methods

Framework for mapping effect pathways

We developed a framework shown in Figure 1 of the potential pathways for how loans from external official creditors and ensuing debt repayments can affect government health spending. The framework was developed iteratively based on the available literature, economic theory, and our study findings. We used the framework as the foundation for analysing and interpreting our data.

[Figure 1 here]

Figure 1: Framework illustrating how lending activities from external official creditors may affect domestic government health spending.

External official creditors give loans to the recipient government as general budget support or earmarked funds to specific sectors, including the health sector. Over time, debt plus its interest accumulates and needs repayment from the general government budget. Government health expenditure budget is also determined by levels of domestic resource mobilisation, development assistance, and political prioritisation. Domestic resource mobilisation relies in part on the amount of taxable economic activity, which is co-determined by external official lending that may help grow the economy. This results in a question of relative balance: Which sectors benefit more from development lending, and which sectors are "picking up the tab" for repaying investments in other sectors? Importantly, the debt burden effects will depend on the terms of lending, and concessional loans will result in a lower debt servicing burden than non-concessional loans, all things equal. The degree of concessionality depends on the grant element of a loan and is defined by the Organisation for Economic Cooperation and Development (OECD) Development Assistance Committee (DAC) as a measure of credit softness reflecting "*the benefit to the borrower compared to a loan at market rate*" [39].

For the health sector, these relationships may help determine how much of the government budget is allocated to the health sector. A larger government health budget may be able to reach more people and cover more services, which can result in a lower degree of reliance on

user fees and other private health financing mechanisms in the health system, forming the financial foundation for Universal Health Coverage (UHC) [40].

Study setting

Situated in Francophone West Africa, Senegal has been one of the most stable democracies in the region, and in Africa more broadly [41], except for political turmoil that erupted over former President Macky Sall's attempt to postpone the 2024 presidential elections [42]. The country became independent from French colonial rule in 1960 [41]. In 2023, the country had a population of approximately 18 million [43] and a GDP per capita of $1,706 in 2023 [3], resulting in a WB classification as a Lower Middle-income Country (LMC). We chose Senegal as a case country for four main reasons. A: Its political stability at the time of fieldwork, so our results were reflective of creditor-government dynamics under stable conditions. B: Its relatively low perceived corruption levels (2023 and earlier) to minimise the risk of changes in health financing occurring due to corruption and not the factors of interest to our study [44]. C: A growing public external debt burden[2] [3] (Figure 2 and Figure 3). D: A health financing composition increasingly characterized by user fees and less so by government contributions at the time our interviews were performed in 2019/20 [9] (Figure 6), inviting questions as to the determinants of this trend.

The appendix (A.1) contains additional information on the health system organisation in Senegal.

Study design, data collection, management and analysis

Study approach

This study was a mixed-methods case study comprised of descriptive statistical analysis of health financing contributions in Senegal, key stakeholder interviews and purposive document review. The analysis of quantitative data and document analysis were performed after the initial analysis of interview data, going back and forth between all sources of data while writing up the results.

We considered the following as evidence of external official lending and public external debt obligations influencing government health spending. A: Verbal statements from interviewees identifying a such influence or effect; B: written documentation of a such, or C: discernible patterns between levels and changes in external official lending, public external debt servicing and government health spending.

[2] In 2017, the Senegalese government changed its definition of public debt to also include parastatals, state-owned enterprises and central government issued guarantees, resulting in an increase in public debt per GDP of about 11% [45].

Quantitative data analysis

We analysed data on Public and Publicly Guaranteed (PPG) loan disbursements in Senegal separated by creditor type and PPG external debt stock and service from the WB [3, 7]; government revenue and expenditure data from the IMF and WB [3, 46]; total official development loans received by sector from the OECD [47]; and GHE-S, External health financing (EXT), OOP and Voluntary Health Insurance (VHI) from the World Health Organization (WHO) [9].

Data was used as historically available in databases. WB data on public borrowing and debt were available from 1970-2023; IMF/WB data on government revenue and expenditure from 1994-2022; OECD Creditor Reporting System Official Development Assistance (ODA) data from 2002-2023; and WHO data on health financing from 2000-2022. All dollar-values were deflated into 2023 constant US$ using WB US$ GDP deflator data [3].

We analysed and presented the quantitative data as trends over time, comparing changes in public borrowing, debt burden and the levels and balance of different health financing sources over time, as well as the overall sectoral distribution of development lending in Senegal. This was done to A: examine the extent to which the health sector has benefited from development loans; B: explore associations between debt servicing levels and government health spending, and C: add context to and triangulate findings from other sources. This was not done to directly establish causality, but rather to generate hypotheses and look for evidence supporting or challenging findings from other data sources.

For this analysis, the general hypothesis was applied of an inverse relationship between debt servicing and government health spending due to a general budget constraint. For comparison, we also explored levels and changes in government education spending to look for evidence of debt servicing impacting across social sectors or differentially between them, using data from the WB [3].

Interviews

We first mapped key stakeholders including relevant donor- and creditor representatives, government representatives, academics, civil society leaders and hospital management staff. We then used snowball sampling to identify additional key informants. A total of 32 stakeholders were identified for interview of which 25 agreed to participate. Interviews were conducted in Dakar between September 30, 2019 – January 31, 2020.

Our final sample included representatives of four bilateral development partners, four multilateral development partners, 9 from central government across four different ministries,

four from Senegalese civil society organisations, three from academia and one from hospital management.

We used the Semi-Structured Interview (SSI) method, allowing the interviewer ([illegible]) to probe and explore interviewee responses in further details as relevant. Interviews were conducted in French or English, using an interpreter for interviews in French. Interviews were recorded and transcribed by a professional transcriptionist, where consent was given for this. The participant information sheet used, consent form and an example interview topic guide can be found in the online LSHTM data repository [48] and the Online Appendix (A.1).

The interview data were analysed using the Framework method [49]. Themes were established deductively based on the research questions as well as inductively from the interviews. The thematic coding framework was validated by an independent researcher before coding all interview data, using NVivo 12 [50]. Participant views and key quotes were summarised across themes in a Framework matrix [49, 51]. Identified patterns in these summary findings were then integrated into the results section. All interviewee statements were anonymised when writing up the paper.

Document review

We searched Senegalese government websites [52-57], and the Senegalese websites of the International Monetary Fund (IMF) [58, 59] and the World Bank (WB) [60] for documents discussing an influence of public external lending on government health spending (215 full text documents reviewed). We also searched across all major academic publishing databases, using the London School of Hygiene & Tropical Medicine Discover platform and Google Scholar, looking for academic literature describing external official lending or public external debt in Senegal, including its effects on health spending (no time constraint, see appendix A.1 for search strings used). Ten full-text academic articles were retrieved for review. Our inclusion criteria for the results section was that documents/articles discussed effects of lending on government health spending in Senegal. The document review was carried out in stages from the time interviews were conducted in 2019/20 up until March 2025.

Ethics statement

We obtained ethical approval from the London School of Hygiene Observational / Interventions Research Ethics Committee (*ref: 16420*) and the National Health Research Ethics Committee in Senegal (*protocol SEN19/56, ref:* 00172). Formal written consent was obtained from all study participants.

Results

Trends in borrowing and debt

In this section, we illustrate how Senegal's PPG external debt stock and servicing have strongly exceeded average levels for Sub-Saharan Africa (SSA), Low Income Countries (LICs) and Lower-Middle Income Countries (LMCs) over the past ten years (Figure 2). We also show how real-term public borrowing in Senegal has increased substantially since 2000, along with overall government revenue, expenditure and GDP. We find that GHE-S has not followed the economic expansion seen, instead showing limited growth over time.

Between 1970-2023, Senegal has borrowed approximately US$48.2 billion from its creditors (constant 2023 US$)[3] [7]. Of this, approximately US$12.2 billion has been borrowed from bilateral creditors, US$9.6 billion from the WB's concessional IDA branch, US$8.7 billion from other multilateral creditors, US$6.6 billion via bonds, US$5.9 billion from commercial banks and other private creditors, US$4.8 billion from the IMF, and $0.4 billion from the WB International Bank for Reconstruction and Development (IBRD) branch[4] [3].

As shown in Figure 3, Senegalese public borrowing increased during the 1970's, leading to debt buildup, which was partly relieved in the Heavily Indebted Poor Countries initiative, providing 850 million US$ in debt relief from 2000-2004 [61]. Since 2000, Senegalese annual borrowing levels from foreign creditors have risen substantially, up from approximately $0.3 billion in 2000 to $3.5 billion in 2023 (constant 2023 US$) [3]. Increases were most pronounced from 2016 and onward, in part due to the government's issuance of US$5.4 billion worth of bonds (Eurobonds) in separate tranches, beginning in 2009 [3, 62-64] (Figure 3). By 2023, Senegal owed a total US$19.2 billion to its creditors [3]. Of this, $4.2 billion was owed to bondholders, US$4.4 billion to the WB IDA, $3.3 billion to other multilateral creditors, $3.1 billion to bilateral creditors, $2.3 billion to the IMF, and $2.0 billion to commercial banks and other private creditors [3, 7] (Figure 3).

The PPG external debt of Senegal corresponded to 55% of its GDP in 2023, well above the average for other LMCs (16%) and SSA countries (25%) (Figure 2) [3]. In 2021, real-term PPG external debt servicing peaked at US$1.6 billion (constant 2023 US$), corresponding to 20%

---

[3] *The used IMF lending data includes drawings made on the IMF general resource account, not the reserve tranche, for which we were unable to find a data source. IMF disbursements and resulting outstanding credit are not counted in PPG external debt figures and were added to attain the aggregate numbers presented in this section*

[4] *Some of the amount borrowed via bonds may be double counted in the other categories, as these creditor types may buy government bonds, however we were unable to identify more detailed data on the holders of Senegalese bonds to be able to avoid this.*

of GGE, outgrowing GHE-S by a factor of 4.3 [3, 9, 46]. While having subsequently decreased, it has done so in proportion to GHE-S which also decreased, maintaining a factor of 4.1 compared to GHE-S in 2022 (Figure 3).

[Figure 2 here]

Figure 2: Public and Publicly Guaranteed (PPG) External debt stocks (panel A) and service (panel B) as fractions of Gross Domestic Product (GDP) in Senegal compared to Low- and Middle-Income Countries (LMICs) and Sub-Saharan Africa (SSA) (%), 2000-2023 [3]. SSA grouping is with high income countries excluded. LICs: Low Income Countries. LMCs: Lower Middle-income Countries. UMICs: Upper Middle-Income Countries.

[Figure 3 here]

Figure 3: ***Panel A****: Public and Publicly Guaranteed (PPG) borrowing in Senegal separated by creditor type, 1970-2023 (billion constant 2023 US$) [3, 7]. Data are for disbursements, i.e. transfers made in each year (flows, as opposed to stock in Panel B). IMF loan disbursement data is only available for drawings made on the IMF general resource account, not the reserve tranche.* ***Panel B****: PPG debt stock in Senegal from 1970-2023 [7]. IMF credit includes all IMF loans except amounts drawn against the country's reserve tranche position. It includes Special Drawing Rights (SDR) allocations [7]. Note: There may be some overlap between "Bonds" and the other creditor categories as these may buy Senegalese bonds, however we were unable to identify more detailed data on the owners of Senegalese bonds to be able to avoid double counting.* ***Panel C****: Gross Domestic Product (GDP) (1970-2023), PPG external debt stock (1970-2023), and General Government Expenditure (GGE) in Senegal (1994-2022) [3, 46].* ***Panel D****: PPG external debt service (1970-2023) and Government Health Expenditure as a Source (GHE-S) (2000-2022) in Senegal [3, 9]. (Data deflated using WB US$ GDP deflator [3]. Note: PPG external debt does not include the use of IMF credit [7]).*

Has lending directly benefited the health sector?

Figure 4 shows the distribution of development loans received in Senegal between 2002-23 by sector [47]. Health and population and reproductive health have received approximately 7% of all development loans, compared to 32% to economic sectors, 11% to production sectors, and 6% to the education sector [47].

[Figure 4 here]

Figure 4: Total official development loans received by sector, Senegal, 2002-23 [47]. Data deflated using US$ GDP deflator [3].

Although not the primary focus of ODA lending, the observation can be made that direct benefit to the health sector from ODA lending has increased over time. This has occurred as a growing amount of ODA has been disbursed for health (Figure 6), of which a growing share has been disbursed as loans [47] (Appendix A.2). In 2023, 33% of ODA for health was disbursed as loans, compared to 3% in 2015, following a general trend for ODA across all sectors in Senegal [47] (Appendix A.2). This expansion has not occurred at the expense of ODA grants

for health, which were US$170 million in 2015 and US$163 million in 2023 (constant 2023 US$) [47].

Several interviewees across stakeholder groups however correctly believed that external official loans predominantly went to economically profitable sectors (natural resources, production, manufacturing etc.), and not social sectors. The government and donors were explained as being jointly interested in ensuring a return on investment:

"I think lending goes more into the economic sectors, and the health and social sectors are less important… The donors or partners who finance … expect a return on investment. They have not invested much in the social, the social is not economically profitable, so many partners prefer to invest in the economic than in the social." (CSO)

A government interviewee concurred that they encouraged external official loans where these could be profitable:

"… Now more and more, we still want to guide partners to finance loans especially in areas that can be profitable". (Government)

Has lending indirectly benefited the health sector?

Lending to the economic and production sectors could have also indirectly supported increases in GHE-S by strengthening the foundation for raising revenue for the public budget, some of which could be spent on health. As development lending has increased (in itself a source of government revenue) (Figure 4), Senegalese government revenue has expanded from US$1.5 billion in 2000 to US$5.7 billion in 2023 (constant 2023 US$) (Figure 5) [46]. Senegalese GGE has grown from US$1.4 billion in 2000 to US$7.6 billion in 2022, while GDP has grown from $10 billion in 2000 to $31 billion in 2023 (constant 2023 US$) (Figure 3 and Figure 5) [3, 46]. Against this economic expansion, GH[illegible]S has however decreased substantially as a share of GGE, from 9.0% in 2000 to 3.4% in 2022 (Figure 5) [3, 9]. In real terms, this translates into a decrease from $300 million in 2006 to $257 million in 2022 (constant 2023 US$), or from $26 per capita to $15 per capita (Figure 5 and Figure 6)[5] [3, 9]. This real-term decrease in GHE-S does not lend support to the hypothesis of a positive indirect effect of lending to other sectors, although the counterfactual trend in the absence of lending is unknown.

[Figure 5 here]

Figure 5: Panel A: General Government Expenditure (GGE) and government revenue as a proportion of GDP and Government Health Expenditure as a Source (GHE-S) as a proportion of GGE in Senegal between 2000-2022 [9, 46]. Panel B: GHE-S from 2000-2022 in Senegal [9]. (Data deflated using WB US$ GDP deflator [3].

[5] *Numbers deflated using US$ GDP deflator data for consistency across figures*.

Has debt servicing constrained government health spending?

Our interview and document review findings point to a constraining effect of public debt servicing on government health spending. Government prioritisation of servicing its debt obligations was perceived by some interviewees as receiving higher priority than funding the health sector, along with making profitable investments to ensure economic growth. The education sector however appeared to suffer less from a debt servicing constraint than the health sector, possibly due to receiving higher government priority.

As seen in Figure 6, real-term OOP per capita expanded considerably in the late 2000s to remain the predominant source of health financing in Senegal. GHE-S has conversely decreased per capita since 2006 to US$15 in 2022 (constant 2023 US$), some of which has been picked up by development assistance, while Social Health Insurance (SHI) and Voluntary Health Insurance (VHI) remain at much lower levels (both less than $5 per capita in 2022 (constant 2023 US$)) [3, 9].

[Figure 6 here]

Figure 6: Health financing per capita by source in Senegal from 2000 to 2022 (constant 2023 US$) [3, 9]. OOP: Out Of Pocket payments. Dom. gov. trans: Transfers from domestic government revenue allocated to health purposes. EXT: External health financing. Vol. pre: Voluntary prepayments. SHI: Social health insurance. Government subsidies to SHI are deducted from SHI figures. Data deflated using US$ GDP deflator data.

For our observational time series data, no pattern was identified between year-on-year changes in PPG external debt servicing and total government current health spending (Appendix A.2) [3, 9]. A negative relationship between debt servicing and government health spending was however identified by many interviewees (below). This indicates that the fiscal reality experienced by key informants may not easily be captured simply by observing year-to-year changes in debt servicing and health spending levels.

When comparing the two main social sectors, total government education expenditure has been consistently higher than total government health expenditure, and increasing in real terms over time (domestic and external source, including capital expenditure) (Appendix A.2) [3, 9]. In 2021, total government education expenditure was 2-2.6 times total government health expenditure (Appendix A.2) [3, 9]. ODA for health and for education have been at comparable levels since 2002 [47], and differences in ODA therefore do not explain the differences in government spending.

Across SSA, education sectors do receive more government funds than health sectors on average [3, 9], however with a considerably lower ratio than in Senegal at about 1.3-1.4 on

average (15.6% of GGE for education compared to 11.1-12.0% for health) (2021)[6] [3, 9]. The Senegalese government thus appears to have given relative priority to the education sector, and any constraining effects of debt servicing present seem to still have allowed the government to provide significantly more and increasing levels of education spending compared to health spending.

An academic explained that the government prioritised the education sector above debt servicing and the health sector below both:

"*Health is* [number] *7 … in the hierarchy of priorities in Senegal … 1 is education, 2nd is roads, the third is debt payment*." (Academic)

This would help explain why the education budget would be less likely to be constrained by debt servicing requirements than the health sector as a result of government prioritisation.

Approximately half of participants, representing all stakeholder groups, believed that Senegal's debt constrained the public budget, including the health budget. Some did not believe that this was the case, while others were not knowledgeable on the matter.

Several interviewees across all stakeholder groups believed a constraining effect of Senegal's debt to impact across all sectors. A government official explained how the health sector was not exempt, and the government at times had to cut them to meet debt obligations.

"We live it every day… Because we are obliged to pay the debt … we are obliged to make budgetary regulations on such sensitive sectors as health, education, even though we try to spare them." (Government)

A donor concurred:

"Absolutely, [debt obligations are] *going to affect the health sector because we know that these loans … the government of Senegal will have to respect their engagement in terms of timeline. If during this time … they have to pay back these loans, they don't have enough resources, they have to cut somewhere. And when they cut, the health sector is also affected."* (Donor)

The cited donor believed that IMF fiscal objectives compounded the effect of the debt on social sectors:

[6] *Both education and health figures include domestic government current and capital spending, and external transfers via government. The lower bound for the ratio includes all external capital health spending as well, while the upper bound does not, as it is not specified whether these funds are distributed via government or not. To balance the risk of bias from measurement error versus sample representativeness, SSA countries with entire variables missing were removed for these calculations (n=9), resulting in 40 remaining countries, some of which had individual values missing for some variables, in particular for capital health spending (n=23).*

"*The IMF sets an objective to the government in term of what should be the level of debt and deficit each year. And so … the government needs to adjust its budget accordingly and so at the end of the day it's the budget for social sectors that are cut*." (Donor).

This interviewee did however attribute this mainly to the government and highlighted the change in attitude from the WB toward prioritising social sectors.

A constraining effect of debt on social spending in Senegal has been acknowledged in official country reports both when debt levels were highest around the turn of the millennium, and again in 2020. The 1998 Enhanced Structural Adjustment Facility policy framework paper recognised that "*the fiscal burden* [of external debt] *remains high, however, with debt service to revenues projected to be 38 percent in 1998. This makes it more difficult to increase expenditures, including for the priority sectors of education and health"* [65]. In the 2002 Poverty Reduction Strategy Paper (PRSP), it was similarly stated that: "*the country's debt burden is a major obstacle to efficient allocation of public resources in favour of the social sectors*" [66]. In the recent context of Senegal's growing debt burden, a 2020 IMF country report for Senegal stated that: *"… debt service continues to absorb a sizable portion of fiscal revenues, thus limiting room for other expenditures in critical areas such as infrastructure investment, health, and education"* [67].

Factors compounding debt servicing constraint of government health spending

A few interviewees highlighted certain government prioritisations made under budget constraint, driving spending away from the health sector, some of which were thought to be exacerbated by debt servicing obligations. This included a focus on sectoral visibility, profitability, a belief in economic growth as first priority before social sectors, and cutting the health sector due to the presence of donors.

Firstly, an increased focus on sectoral visibility due to a debt servicing constraint on the government budget was explained by a CSO representative as politicians focusing spending where it would help secure re-election, when there was little left to spend:

"If the money mobilized to repay the debt … returns to the donors, the government which does not have much means will prioritize … and they are not going to put it into health. They will put it in another much more visible sector to say that "I am building an airport, I am building roads". … I have never seen a politician during an election campaign say that "I treated so many people" … They say: … "I don't have a lot of money. The little I have, I put it in the sectors that allow me to be re-elected in the next elections". (CSO)

Secondly, lack of profitability of the health sector was also believed by the same interviewee to be an explanation for why the sector received less government priority and was subject to budget constraint from debt servicing obligations:

Q: "Do you think the health sector is more affected than other sectors in terms of being constrained to pay off debt?"

A: "*Yes I think so … As I said, health, the social, is not profitable. This is why we do not prioritize it.*" (CSO)

Another CSO leader concurred that health and social protection investments received last priority among government expenses, exacerbated by the weight of the debt. They described the conditions and priorities of the ministry of the budget as follows:

From [the small] *budgetary space you must first pay the debt, after that the security, the sovereign expenses, the operations, before being able to give water and health to the populations … The weight of the debt does not allow to invest in basic social services.*" (CSO).

Thirdly, a donor identified a dynamic in which the presence of donors to step in and cover costs in the health sector rendered this sector more likely to be subject to budget cuts during the financial year to meet debt obligations:

"[Cuts due to debt obligations] *can affect any sector, but specifically for the health sector, and the reason it's going to affect more the health sector is the government knows that the health sector will receive more donor resources compared to the others"* (Donor).

Discussion

With this study, we aimed to unpack how external official lending and debt have affected government health spending in Senegal. Our findings indicate that Senegal's debt has constrained the general government expenditure budget and thereby the health sector budget. This effect has been mitigated by general revenue expansion through development loans received, which have also directly benefited the health sector. Unexpectedly, the education sector appeared to have been insulated from debt effects due to a higher political prioritisation. The majority of our study participants who held knowledge on the matter, across all stakeholder groups, believed that Senegal's debt burden constrained the public budget, including the health budget. Official IMF and WB documents from 1998, 2002 and 2020 supported this assessment [65-67]. Given the consistent growth of Senegalese GGE, this finding should be interpreted as a relative consideration of the public expenditure growth rate, and the counterfactual of GGE in the absence of external official lending is not known. GHE-S however decreased as a share of GGE and did not grow in real, absolute terms between 2006 and 2022 (using US$ GDP deflator [3]). This decoupling deviates from findings across 133 LMICs, showing that around 70% of the budgetary space for health is driven by changes

in overall government expenditure, with the remaining 30% being attributable to the expenditure budget share allocation to the health sector [68]. In Senegal, the health sector's share allocation has decreased so much that any effects from growth in GDP, revenue and expenditure have been largely cancelled out.

Some of our interviewees helped explain these findings by saying that the government prioritised economic growth and servicing its external debt before funding the health sector. The health sector was also described as a sector with relatively low visibility and political value around elections. These political economy factors elicited via qualitative enquiry constitute "country specific features" that co-determine limited health budget prioritisation beyond statistical measures such as national income or perceived corruption [69]. In Ghana, where GHE-S is much higher per GGE and per capita, the performance of the National Health Insurance Scheme has been central to the fate of political parties around presidential elections [9, 70]. In Nigeria however, where funding levels are comparable to Senegal, self-reinforcing beliefs of the superiority of the private health sector over an underfunded public health sector have been pervasive [9, 71]. Further qualitative research building on studies such as [70, 71] that directly seek to identify the most effective levers and arguments for strengthening political commitment to health sectors in Senegal and other parts of the ECOWAS region are needed to help promote progress on the Abuja pledge of 15% of the government budget being allocated to the health sector [72].

If the cycle continues of lending primarily for economic growth and meeting increasing debt obligations before investing in the health sector, the outlook for a revitalisation of the budget priority given to the health sector in Senegal appears limited. While investments in the highly prioritised education sector could help support economic growth and thereby the revenue basis for GHE-S, such synergies were not reflected in trends in GHE-S over time in the period of study. They could however materialise in the future, provided GHE-S receives increasing budget priority out of GGE.

Our finding of debt constraint of the health budget extends some previous findings by other authors. Two studies of the period 1970-2000 found that the Senegalese government spent around 20 percent of its resources on debt servicing [73, 74], and that a one percent increase in debt servicing was associated with a reduction in total domestic expenditure by 0.13 percent of GDP [73, 74]. With concerns of future debt crisis in Senegal, the identified past effects could become worse over time [38]. Our findings also align well with existing case studies from Kenya [29, 30], and with multi-country econometric studies, generally finding negative associations between debt variables and government health spending [19-26, 28] (one study has had more variable findings [27]). By taking a mixed methods approach with new primary data generation in a new country context through key informant interviews, combined with in-depth secondary data exploration, our study was able to expand on the existing literature by

identifying and unpacking the fiscal implications on health spending from external lending at the country level. Beyond the identified debt constraint, our study also provided empirical evidence for the expected though essential point that loans may benefit the health sector in the first place. This is important for a more nuanced understanding of the full life-cycle impacts of development lending on health financing. The more the portfolio of government development loans are directed toward the health sector in a sustained manner over time, the less likely they are to cause a net erosion of the government health budget when loan repayments are taken into account (adjusted for inflation and value discounting). This is moderated by the revenue-expanding effects of development lending for economic growth, provided the added revenue benefits the health sector, which we did not find evidence for in Senegal in the period of study.

Importantly, by showing how government education spending kept on growing in spite of debt obligations as opposed to health spending, our study provided an original empirical basis for the interpretation that a constraining effect of debt on spending in a given social sector may be modified or even fully negated by political priority of that sector. This is a novel finding which leads to policy recommendations as seen in the conclusions section. It deviates from most of the reviewed econometric literature on public debt and education spending, mostly finding a negative link [19, 20, 31-37]. This illustrates the importance of an appreciation of the political economy and fiscal prioritisations made at the individual country level, and the challenges of extrapolating findings from the cross-country level[7] to the individual country.

Additionally, it deserves mention that while this paper focused on the modality of development lending, we acknowledge the important EDP contribution of providing grant financing. In spite of its decreasing share over time, it has provided 81% of ODA for the health sector in Senegal on average between 2002-2023 [47]. The significant overall ODA funding flows to the health sector in Senegal offer another explanation for why this sector has not been prioritised by the Senegalese government through aid displacement of government funds (fungibility) [26, 28, 75-88]. This has the same negative direction of effect on health financing as the identified debt constraint, constituting a dual challenge for revitalising health spending in Senegal, and potentially in other countries experiencing similar fiscal conditions.

Finally, the recent cuts to ODA from the United States, France, Germany and other donors will leave a funding shortfall across sectors in Senegal [89, 90]. To mitigate this and avoid adverse consequences for the Senegalese people, other partners and/or the Senegalese government will have to step in and cover costs. The latter adds the risk of necessitating

[7] [32] and [35] were single-country studies.

external borrowing, which could further fuel downstream debt constraints on the health sector, in turn strengthening the case for debt relief in Senegal (see Conclusions section).

Limitations

Unpacking our macroeconomic and fiscal dynamics of interest is inherently complicated, with complex causal chains, numerous contemporaneous determinants and competing political considerations. This served as the motivation for our multipronged methodological approach and led us to focus more on identifying and discussing different pathways of effect while remaining cautious about drawing causal inferences.

Using descriptive statistics based on relatively short observational time series to quantitatively assess sectoral impacts from cross-sector fiscal level burdens proved challenging. This is an inherent statistical limitation when using single-country observational macroeconomic data, further complicated by the fact that debt impacts the general public budget before sectoral allocations. Using complementary data sources such as individual stakeholder accounts or written reports were necessary to discuss possible causal relationships.

Importantly, a series of audits have revealed that Senegal's public debt had been underreported by the previous administration, adjusting debt-to-GDP estimates up to a critical 119% by end-2024 [91-93]. In light hereof, our quantitative findings for Senegal's debt burden may serve as a lower bound, its impacts on government health spending may be even larger than our findings suggest, and these may become worse over time unless the debt burden is addressed.

In interviews, five participants did not consent to recording of the interview or quoting them, so instead FF took notes and used these interviews to broadly further his understanding of the research questions and the interviewee's position in regard hereto. Also, potential interviewees in one government agency, two hospitals, two multilateral organisations and two academics did not respond or were unavailable. However, our interview group for this study was overall well balanced, with 8 development partners, 9 government representatives, and 8 non-government representatives.

Conclusions and future research

This research has added to a limited set of case studies of the impacts of debt on government health spending in SSA. We were able to significantly expand on the existing literature in

particular in eliciting key stakeholder accounts of the fiscal reality in Senegal, and providing a full view of influence from initial lending to debt servicing obligations, the first of which is often neglected in the literature. The identified relative insulation of the education sector from debt servicing obligations due to political priority illustrates how quantitative studies grouping all social sector spending as a single outcome variable may miss important sectoral effects. These nuances are also important in current and coming debates on debt relief, if the intention is that any funds freed up should preferentially support struggling social sectors and not follow the existing fiscal budget prioritisations in a debtor country.

While development lending has benefited the health sector in Senegal, the health sector should be protected from unprecedented public external debt servicing levels. This responsibility falls both on domestic policy makers, EDPs and the IMF. Enough flexibility should be made in repayment timelines to avoid the government having to consider cutting social sectors in order to make repayments. Exploring options for debt relief to address the rapidly growing debt burden of Senegal may become necessary, however our findings indicate that lower debt servicing levels do not equate increased health spending without political priority. Mechanisms such as debt-to-health swaps could be considered to ensure the health sector would benefit from any funds made available through debt relief [23]. This could also help level out the persistent and growing imbalance between the health and education sectors seen and prevent the health sector from losing out behind what is perceived as more economically profitable sectors.

As long as economic growth and servicing its now historical debt levels are top priorities for the Senegalese government to meet its obligations to EDPs, the IMF and private creditors before investing in the health sector, substantive real-term GHE-S gains and expansion of public-sector driven UHC expansion appear unlikely. Supported by debt relief, increased government priority of the health sector will be necessary to break this cycle, working toward the Abuja pledge of 15% of the government budget being allocated to the health sector [72] - ideally from domestic revenue. This kind of fiscal policy turnaround may be a necessary precondition for sustained progress on UHC in Senegal. In advocacy work and discussions with the Senegalese government, using the substantial literature that establishes health as a key driver of economic productivity might be helpful for making the case for increased health investments (e.g. [94]).

Additionally, recent ODA cuts from several donors may necessitate further external borrowing to cover costs. This strengthens the case for A: relief of existing debt obligations, and B:

increasing the availability of concessional grant and loan financing from other partners, in order to avert unsustainable debt levels and exacerbated constraint on the health sector.

Future research avenues include exploring the lending and debt implications for the health sector in other countries using qualitative and mixed methods to understand individual country realities. Such case studies could focus on countries at high risk of or in debt distress, also exploring how implications of debt translate at different levels of the health system. Countries may respond and prioritise differently when burdened by debt, and understanding individual country impacts may be important for informing both creditor policy to avoid averse social impacts, and to inform a redistribution of any freed-up funds in future debt resolution processes toward sectors and programs that have been heavily impacted by debt.

Importantly though, keeping in mind the range of beneficial effects of concessional development lending, which had also benefited the health sector in Senegal, is essential for a nuanced and complete understanding of the health financing implications of development lending.

In Senegal, the determinants underlying the relative prioritisation of the education sector over the health sector and apparent differential insulation from debt impacts warrants further exploration. This would likely benefit from applying a broad political economy lens encompassing both domestic and external drivers.

Acknowledgements

The authors would like to thank the interview participants for giving us their time and sharing their important insights on the study topic. We are also thankful to Ms. Aminata Diop for interpretation and Ms. Aminta Gueye for transcription of interviews. We thank Dr. Penelope Milsom for helping validate our interview coding framework.

References

1. World Bank. Heavily Indebted Poor Countries (HIPC) Initiative. 2018 [cited 2022 February 3]. Available from: https://www.worldbank.org/en/topic/debt/brief/hipc.
2. Abbasi K. The World Bank and world health: Changing sides. Bmj. 1999;318(7187):865-9.
3. World Bank. World Bank Open Data. 2025 [cited 2025 July 25]. Available from: https://data.worldbank.org/.
4. IMF. Debt Relief Under the Heavily Indebted Poor Countries (HIPC) Initiative. 2021 [cited 2021 October 12]. Available from: https://www.imf.org/en/About/Factsheets/Sheets/2016/08/01/16/11/Debt-Relief-Under-the-Heavily-Indebted-Poor-Countries-Initiative.
5. IMF. Multilateral Debt Relief Initiative — Questions and Answers. 2017 [cited 2021 December 20]. Available from: https://www.imf.org/external/np/exr/mdri/eng/mdrians.htm#q01.
6. Essers D, Cassimon D. Towards HIPC 2.0? Lessons from Past Debt Relief Initiatives for Addressing Current Debt Problems. Journal of Globalization and Development. 2023;13(2):187-231.
7. World Bank. International Debt Statistics. 2024 [cited 2024 April 24]. Available from: https://databank.worldbank.org/source/international-debt-statistics#.
8. World Bank. World Bank Country and Lending Groups. 2024 [cited 2024 April 23]. Available from: https://datahelpdesk.worldbank.org/knowledgebase/articles/906519-world-bank-country-and-lending-groups.

9. WHO. Global Health Expenditure Database. 2024 [cited 2024 March 28]. Available from: http://apps.who.int/nha/database.

10. Kurowski C, Schmidt M, Evans DB, Tandon A, Eozenou PH-V, Cain JS, et al. Government Health Spending Outlook - Projections Through 2029: Diverging Fiscal Pressures, Uneven Constraints. 2024 [cited 2025 August 23]. Available from: https://documents1.worldbank.org/curated/en/099110524145099363/pdf/P506692116ebcb0e188b4175eb4c560cb5.pdf.

11. United Nations Global Crisis Response Group. A world of debt: A growing burden to global prosperity. 2023 [cited 2024 March 25]. Available from: https://www.un.org/regionalcommissionsnyoffice/news/launch-un-report-%E2%80%9C-world-debt%E2%80%9D.

12. World Bank. Health Financing in a Time of Global Shocks: Strong Advance, Early Retreat. 2023 [cited 2025 March 25]. Available from: https://openknowledge.worldbank.org/handle/10986/39864.

13. The L. Economic storms threaten to cast health adrift. Lancet. 2024;403(10424):325.

14. Fan V, Gupta S. What's Rising Debt Got to Do With Health Spending? 2023 [cited 2023 September 27]. Available from: https://www.cgdev.org/blog/whats-rising-debt-got-do-health-spending.

15. OXFAM. No country should be forced to choose between paying back debts or providing health care. 2024 [cited 2024 March 26]. Available from: https://www.oxfam.org/en/no-country-should-be-forced-choose-between-paying-back-debts-or-providing-health-care.

16. Jubilee Debt Campaign. Sixty-four countries spend more on debt payments than health. 2020 [cited 2025 November 24]. Available from: https://debtjustice.org.uk/press-release/sixty-four-countries-spend-more-on-debt-payments-than-health.

17. Amnesty International. Debt and the Right to Health: Closing Observations from Our Series. 2024 [cited 2024 March 26]. Available from: https://www.amnesty.org/en/latest/campaigns/2023/10/debt-and-the-right-to-health-closing-observations-from-our-series/.

18. Development Finance International. The worst ever global debt crisis. 2023 [cited 2024 March 26]. Available from: https://assets.nationbuilder.com/eurodad/pages/3195/attachments/original/1696947958/Debt_Service_Watch_Briefing_Final_Word_EN_0910.pdf?1696947958.

19. Fosu AK. The External Debt-Servicing Constraint and Public Expenditure Composition. Helsinki, Finland: UNU-WIDER; 2007.

20. Fosu AK. The External Debt-Servicing Constraint and Public-Expenditure Composition in Sub-Saharan Africa. African Development Review. 2010;22(3):378-93.

21. Said R, Morai AS. Relationship between Public Debt Burden and Health Expenditure in Sub-Saharan African Countries: The Role of Institutional Quality. Journal of Business and Social Review in Emerging Economies. 2020;6(2):493-502.

22. Chipunza T, Ntsalaze L. Income per capita and government healthcare financing in Sub-Saharan Africa: The moderating effect of indebtedness. Scientific African. 2024;26.

23. Lora E, Olivera M. Public debt and social expenditure: Friends or foes? Emerging Markets Review. 2007;8(4):299-310.

24. Shabbir S, Yasin HM. Implications of Public External Debt for Social Spending: A Case Study of Selected Asian Developing Countries. Lahore Journal of Economics. 2015;20(1):71-103.

25. Lahiani A, Mtibaa A, Gabsi F. Fiscal Consolidation, Social Sector Expenditures and Twin Deficit Hypothesis: Evidence from Emerging and Middle-Income Countries. Comp Econ Stud. 2022;64(4):710-47.

26. Liang LL, Mirelman AJ. Why do some countries spend more for health? An assessment of sociopolitical determinants and international aid for government health expenditures. Social science & medicine (1982). 2014;114:161-8.

27. Behera DK, Dash U. Impact of macro-fiscal determinants on health financing: empirical evidence from low-and middle-income countries. Glob Health Res Policy. 2019;4:21.

28. Patenaude BN. The relationship between development assistance for health and public health financing in 134 countries between 2000 and 2015. Health Policy Plan. 2021;36(4):369-83.

29. Kimalu PK. Debt relief and health care in Kenya. World Institute for Development Economics (UNU-WIDER). 2002;WIDER Discussion Papers, No. 2002/65, ISBN 9291902551, UNU-WIDER, Helsinki

30. Omondi F, Onono PA. Impact of debt servicing on social spending and well being of low-income households in Kenya: Case studies of Nairobi city and Mombasa counties. 2022 [cited 2025 January 16]. Available from: https://kenya.oxfam.org/latest/publications/impact-debt-servicing-social-spending-and-wellbeing-low-income-households-0.

31. Miningou ÉW. External Debt, Fiscal Consolidation, and Government Expenditure on Education. World Bank; 2023.

32. Ekaette SO, Owan VJ, Agbo DI. External Debts and the Financing of Education in Nigeria from 1988–2018: Implication for Effective Educational Management. Journal of Educational Realities. 2019;9(1):55-68.

33. Tilak JBG. External Debt and Public Investment in Education in Sub-Saharan Africa. Journal of Education Finance. 1990;15(4):470-86.

34. Fosu AK. Fiscal Allocation for Education in Sub-Saharan Africa: Implications of the External Debt Service Constraint. World Development. 2007;35(4):702-13.

35. T. K. Public spending on education and external public debt. Government Spending on Education and External Public Debt: Case of Georgia. 2021 [cited 2025 September 10]. Available from: https://ctcskolalab.ge.

36. Mbewe G. Analysis of the Relationship between Public Debt Service and Education Expenditure: A Pooled Mean Group Approach. Asian Research Journal of Arts & Social Sciences. 2024;22(9):95-106.
37. Khundadze T, Alvarez A. Debt mechanisms and education financing: a comparative study in seven countries. 2022 [cited 2025 September 10]. Available from: https://campaignforeducation.org/images/downloads/f1/1857/debt-comparative-paper-final.pdf.
38. Koddenbrock K. Earnest struggles: structural transformation, government finance and the recurrence of debt crisis in Senegal. Review of International Political Economy. 2024;31(6):1788-813.
39. OECD. DAC Glossary of Key Terms and Concepts. 2023 [cited 2023 December 18]. Available from: https://www.oecd.org/dac/dac-glossary.htm.
40. WHO. What are the Overall Principles of HBP Design? 2025 [cited 2025 February 6]. Available from: https://www.who.int/teams/health-financing-and-economics/economic-analysis/health-technology-assessment-and-benefit-package-design/resource-guide-for-the-use-of-hta-and-hbp-design-processes/what-are-the-overall-principles-of-hbp-design.
41. CIA. Senegal. 2022 [cited 2022 January 31]. Available from: https://www.cia.gov/the-world-factbook/countries/senegal/.
42. Amy Niang. 2024 Senegal election crisis points to deeper issues with Macky Sall and his preferred successor. 2024 [cited 2024 April 24]. Available from: https://theconversation.com/2024-senegal-election-crisis-points-to-deeper-issues-with-macky-sall-and-his-preferred-successor-223035.
43. Agence nationale de la statistique et de la demographie (ANSD). Le Sénégal En Bref. 2023 [cited 2023 August 11]. Available from: https://www.ansd.sn/.
44. Transparency International. Corruption Perception Index. 2024 [cited 2024 April 24]. Available from: https://www.transparency.org/en/cpi/2023/index/sen.
45. World Bank. Country partnership framework for the Republic of Senegal for the period FY20-FY24. 2020 [cited 2023 September 18]. Available from: https://documents.worldbank.org/en/publication/documents-reports/documentdetail/608781583719225540/senegal-country-partnership-framework-for-the-period-fy20-fy24.
46. IMF. Public Finances in Modern History. 2024 [cited 2024 June 10]. Available from: https://www.imf.org/external/datamapper/datasets/FPP.
47. OECD. Creditor Reporting System (CRS). 2024 [cited 2023 February 16]. Available from: https://stats.oecd.org/Index.aspx?DataSetCode=CRS1.
48. London School of Hygiene and Tropical Medicine. LSHTM Data Compass. 2025 [cited 2025 August 1]. Available from: https://datacompass.lshtm.ac.uk/id/eprint/3698/.
49. Ritchie J, Spencer L. Qualitative Data Analysis for Applied Policy Research. In: Bryman A, Burgess R, editors. Analyzing Qualitative Data. 1st ed: Routledge; 1994.
50. QSR International Pty Ltd. NVivo qualitative data analysis software. Version 12, 2020.
51. Ward DJ, Furber C, Tierney S, Swallow V. Using Framework Analysis in nursing research: a worked example. J Adv Nurs. 2013;69(11):2423-31.
52. Agence nationale de la statistique et de la demographie (ANSD). ansd.sn. 2024 [cited 2024 July 25]. Available from: https://www.ansd.sn/index.php.
53. Ministère de l'Economie du Plan et de la Coopération. economie.gouv.sn. 2023 [cited 2023 October 10]. Available from: https://www.economie.gouv.sn/.
54. Ministère des Finances et du Budget. Direction de la Dette Publique (DDP). 2023 [cited 2023 October 10]. Available from: https://www.sentresor.org/structures-du-tresor/directions-administratives-da/direction-de-la-dette-publique-ddp/presentation-de-la-ddp/.
55. Ministère des Finances et du Budget. senfinances.org. 2023 [cited 2023 October 10]. Available from: https://www.senfinances.org/.
56. Agence de la Couverture Maladie Universelle. agencecmu.sn. 2024 [cited 2024 July 25]. Available from: https://agencecmu.sn/.
57. Ministère de la Santé et de l'Action Sociale. https://sante.gouv.sn/. 2024 [cited 2024 September 19]. Available from: https://sante.gouv.sn/.
58. IMF. Senegal. 2023 [cited 2023 September 25]. Available from: https://www.imf.org/en/Countries/SEN.
59. IMF. IMF eLIBRARY: Senegal. 2023 [cited 2023 October 10]. Available from: https://www.elibrary.imf.org/subject/SN.
60. World Bank. Open Knowledge Repository. 2023 [cited 2023 October 10]. Available from: https://openknowledge.worldbank.org/.
61. IMF, World Bank. Senegal: Enhanced Heavily Indebted Poor Countries (HIPC) Initiative Completion Point Document. 2004 [cited 2023 September 11]. Available from: https://www.imf.org/en/Publications/CR/Issues/2016/12/30/Senegal-Enhanced-Heavily-Indebted-Poor-Countries-HIPC-Initiative-Completion-Point-Document-17380.
62. OECD Development Co-operation Directorate. A Recipient Perspective on TOSSD: The Case of Senegal. 2017 [cited 2020 November 13]. Available from: http://www.oecd.org/dac/financing-sustainable-development/development-finance-standards/A%20recipient%20perspective%20on%20TOSSD_Senegal.pdf.
63. Wallace P, Pronina L, Ciss M. Senegal Attracts Almost $10 Billion in Eurobond Bids. 2018 [cited 2020 November 13]. Available from: https://www.bloomberg.com/news/articles/2018-03-06/senegal-is-said-to-attract-almost-10-billion-in-eurobond-bids.
64. Reuters. Senegal issues 775 million euros in Eurobonds. 2021 [cited 2023 August 23]. Available from: https://www.reuters.com/article/ozabs-uk-senegal-eurobond-idAFKCN2DF0L7-OZABS.

65. Republic of Senegal, IMF, World Bank. Senegal: Enhanced Structural Adjustment Facility Policy Framework Paper, 1998-2000. 1998 [cited 2023 September 14]. Available from: https://www.imf.org/external/np/pfp/senegal/senge00.htm.

66. Republic of Senegal. Poverty Reduction Strategy Paper. 2002 [cited 2023 September 12]. Available from: https://www.imf.org/external/np/prsp/2002/sen/01/100502.pdf.

67. IMF. Request for a three-year policy coordination instrument—press release; staff report; and statement by the executive director for senegal. 2020 [cited 2023 August 23]. Available from: https://www.imf.org/en/Publications/CR/Issues/2020/01/18/Senegal-Request-for-a-Three-Year-Policy-Coordination-Instrument-Press-Release-Staff-Report-48958.

68. Barroy H, Gupta S. From Overall Fiscal Space to Budgetary Space for Health: Connecting Public Financial Management to Resource Mobilization in the Era of COVID-19. 2020. Available from: https://www.cgdev.org/sites/default/files/overall-fiscal-space-budgetary-space-health-connecting-public-financial-management.pdf.

69. Micah AE, Chen CS, Zlavog BS, Hashimi G, Chapin A, Dieleman JL. Trends and drivers of government health spending in sub-Saharan Africa, 1995–2015. BMJ Global Health. 2019;4(1).

70. Novignon J, Lanko C, Arthur E. Political economy and the pursuit of universal health coverage in Ghana: a case study of the National Health Insurance Scheme. Health Policy Plan. 2021;36(Supplement_1):i14-i21.

71. Chukwuma JN, Obi FA. A political economy analysis of health policymaking in Nigeria: the genesis of the 2014 National Health Act. Health Policy Plan. 2025;40(4):459-70.

72. Organisation of African Unity. Abuja Declaration on HIV/AIDS, Tuberculosis and Other Related Infectious Diseases. Abuja, Nigeria; 2001.

73. Ouattara B. Fiscal Effects of Aid flows in Senegal. 2004 [cited 2023 March 15]. Available from: http://hummedia.manchester.ac.uk/schools/soss/economics/discussionpapers/EDP-0420.pdf.

74. Ouattara B. Aid, Debt and Fiscal Policies in Senegal. Journal of International Development. 2006;18(8):1105-22.

75. Federspiel F, Borghi J, Mbaye EM, Jensen HT, Martinez Alvarez M. Development partner influence on domestic health financing contributions in Senegal: a mixed methods case study. Health Policy Plan. 2024.

76. Mishra P, Newhouse D. Does health aid matter? J Health Econ. 2009;28(4):855-72.

77. Farag M, Nandakumar AK, Wallack SS, Gaumer G, Hodgkin D. Does funding from donors displace government spending for health in developing countries? Health Aff (Millwood). 2009;28(4):1045-55.

78. Stuckler D, Basu S, McKee M. International Monetary Fund and aid displacement. Int J Health Serv. 2011;41(1):67-76.

79. Xu K, Saksena P, Holly A. The determinants of health expenditure: A country-level panel data analysis. Results for Development Institute WHO; 2011.

80. Gebrehanna S, Upadhyay MP. Fungibility of Foreign Aid in Selected African Countries. Indian Development Review. 2012;10(1):63-80.

81. Van de Sijpe N. The fungibility of health aid reconsidered. 2013.

82. Van de Sijpe N. Is Foreign Aid Fungible? Evidence from the Education and Health Sectors. World Bank Economic Review. 2013;27(2):320-56.

83. Fernandes Antunes A, Xu K, James CD, Saksena P, Van de Maele N, Carrin G, et al. General budget support: has it benefited the health sector? Health economics. 2013;22(12):1440-51.

84. Dieleman JL, Graves CM, Hanlon M. The Fungibility of Health Aid: Reconsidering the Reconsidered. Journal of Development Studies. 2013;49(12):1755-62.

85. Dieleman JL, Hanlon M. Measuring the displacement and replacement of government health expenditure. Health Econ. 2014;23(2):129-40.

86. Barkat K, Mrabet Z, Alsamara M. Does Official Development Assistance for Health from Developed Countries Displace Government Health Expenditure in Sub-Saharan Countries? Economics Bulletin. 2016;36(3):1616-35.

87. Younsi M, Chakroun M, Nafla A. Robust analysis of the determinants of healthcare expenditure growth: evidence from panel data for low-, middle- and high-income countries. International Journal of Health Planning and Management. 2016;31(4):580-601.

88. Lu C, Schneider MT, Gubbins P, Leach-Kemon K, Jamison D, Murray CJ. Public financing of health in developing countries: a cross-national systematic analysis. The Lancet. 2010;375(9723):1375-87.

89. O'Sullivan O, Puri J. First USAID closes, then UK cuts aid: what a Western retreat from foreign aid could mean. 2025 [cited 2025 April 10]. Available from: https://www.chathamhouse.org/2025/03/first-usaid-closes-then-uk-cuts-aid-what-western-retreat-foreign-aid-could-mean.

90. Laub K, Setiabudi N, Dwyer S, Barter E, Smole E. The Budget Cuts Tracker. 2025 [cited 2025 April 10]. Available from: https://donortracker.org/publications/budget-cuts-tracker.

91. Reuters. Audit reveals Senegal's debt and deficit higher than reported. 2024 [cited 2024 December 26]. Available from: https://www.reuters.com/world/africa/audit-reveals-senegals-debt-deficit-higher-than-reported-2024-09-26/.

92. Miriri D. Senegal's dollar bonds fall after audit reveals larger debt and deficit. 2024 [cited 2024 December 26]. Available from: https://www.reuters.com/world/africa/senegals-dollar-bonds-fall-after-audit-reveals-larger-debt-deficit-2024-09-27/.

93. Reuters.com. Explainer: Senegal's billions in hidden debt, and why it is an IMF headache. 2025 [cited 2025 September 7]. Available from: https://www.reuters.com/world/africa/senegals-billions-hidden-debt-why-it-is-an-imf-headache-2025-07-24/.
94. Bloom DE, Canning D. Population Health and Economic Growth. 2008 [cited 2025 March 3]. Available from: https://openknowledge.worldbank.org/server/api/core/bitstreams/4e2a9443-7596-5003-88c6-ebc8379a49f7/content.

Figure

Click here to access/download;Figure;figure1_PGPH_ne

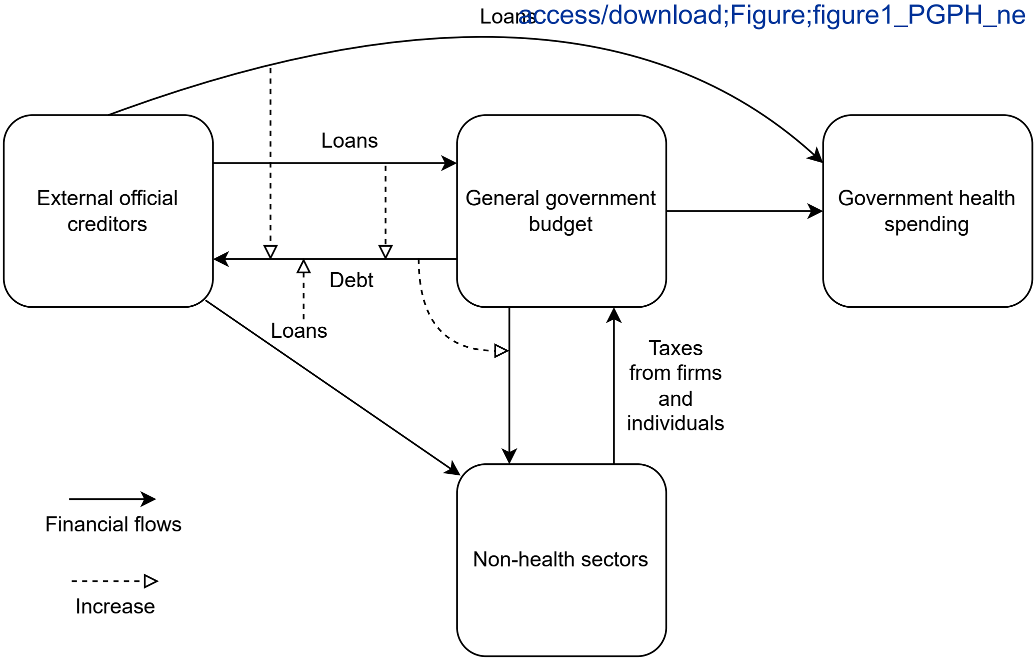

Figure

Click here to access/download;Figure;figure2_PGPH_new.pdf

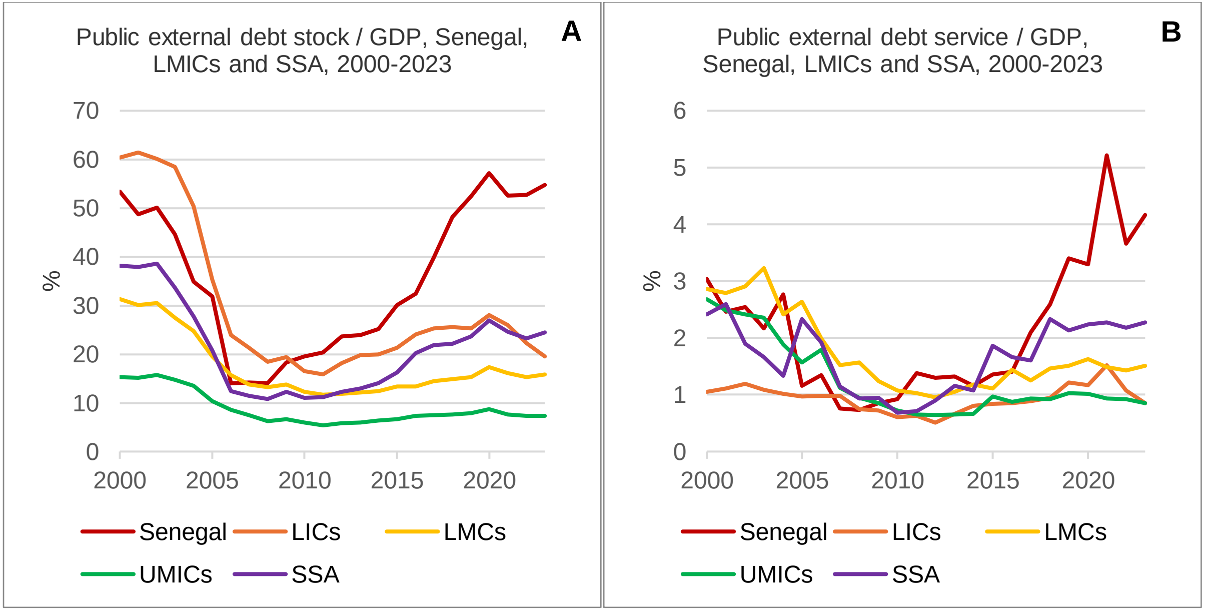

Figure

Click here to access/download;Figure;figure3_PGPH_new.pdf

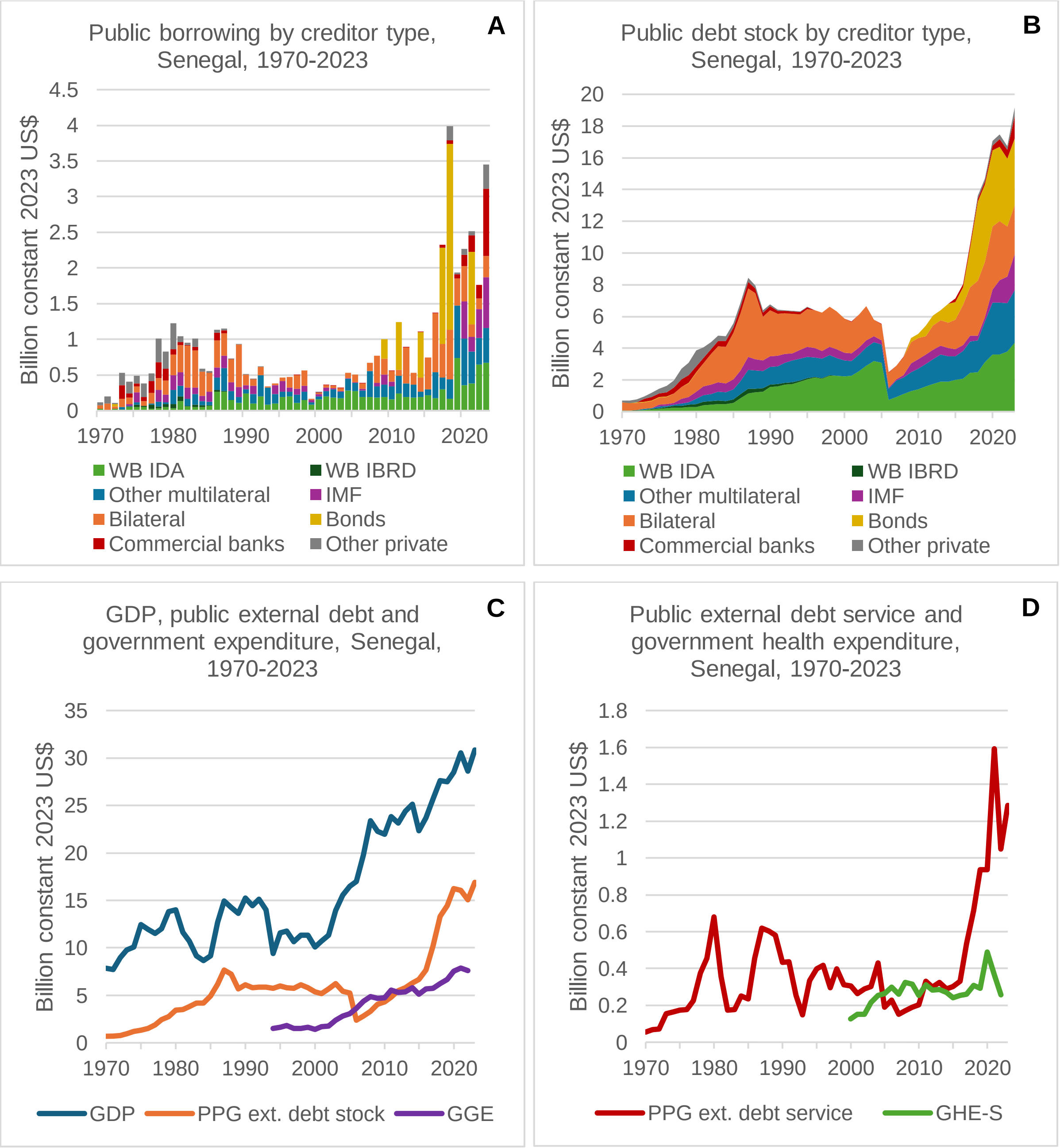

Figure

Click here to access/download;Figure;figure4_PGPH_new.pdf

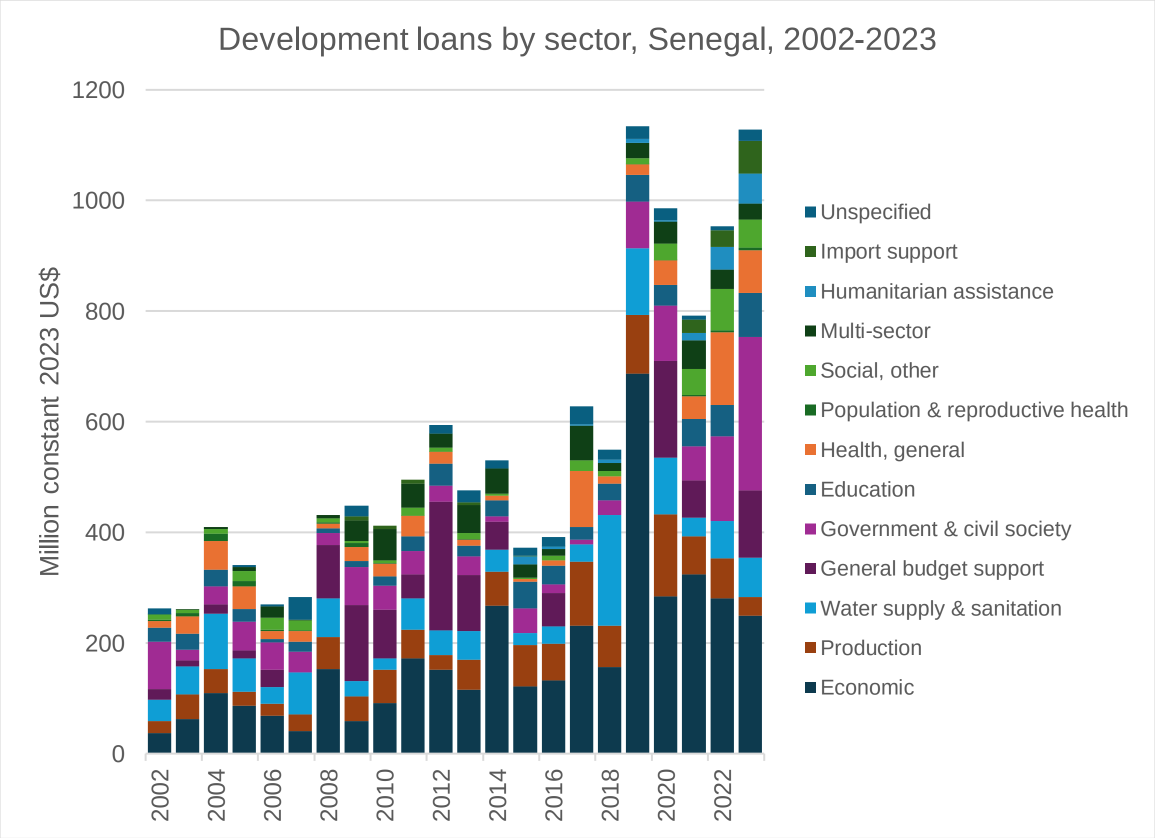

Figure

Click here to access/download;Figure;figure5_PGPH.pdf

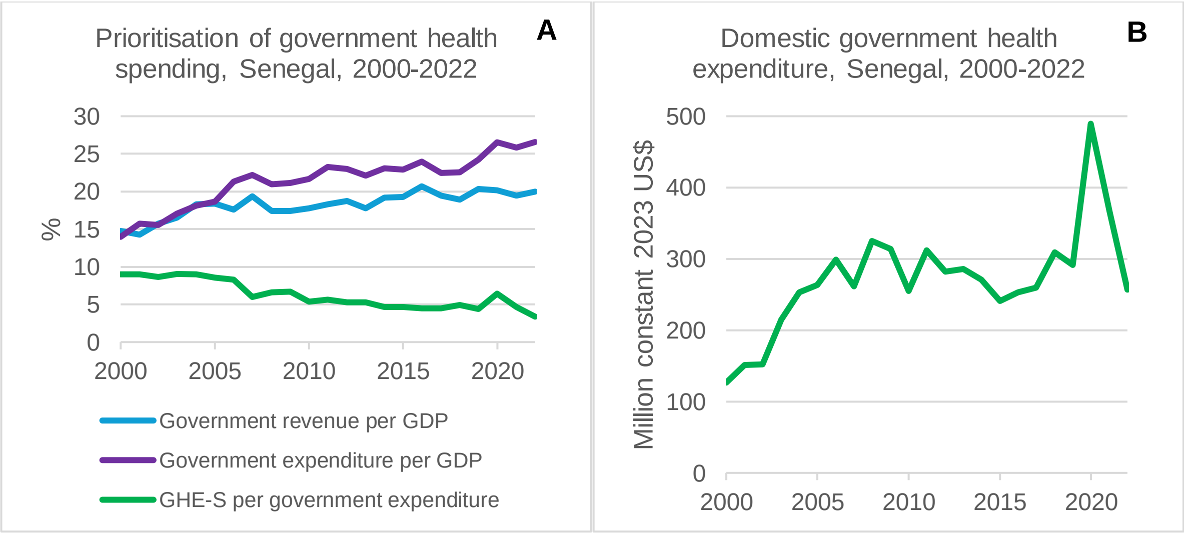

Figure

Click here to access/download;Figure;figure6

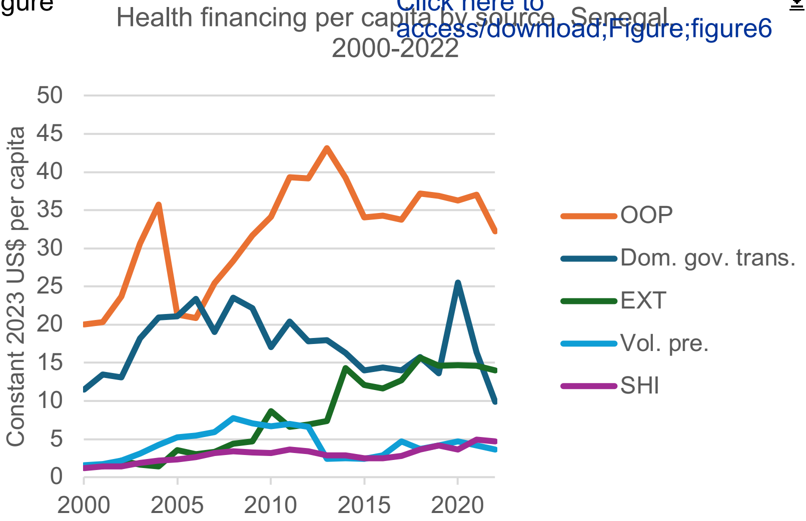

Click here to access/download
Supporting Information
Senegal_debt_healthspending_PGPH_appendix_1.docx
