# Supplementary material for: Influence of public external debt on government health expenditure: A mixed-methods case study of Senegal
Source: PLOS Glob Public Health. 2026 Jul 10;6(7):e0006698. doi: 10.1371/journal.pgph.0006698 (PMC13353989; doi:10.1371/journal.pgph.0006698)
Supplement: Attachment — Submitted filename: debt comment.docx [file pgph.0006698.s003.docx]

Influence of Public External Debt on Government Health Spending: A Mixed-Methods Case Study of Senegal reflects the real-world external debt situation in the developing countries. So it is well done and data results are well documented. I recommend to be published and displayed on websites. Accepted for publication.
