# Supplementary material for: Influence of public external debt on government health expenditure: A mixed-methods case study of Senegal
Source: PLOS Glob Public Health. 2026 Jul 10;6(7):e0006698. doi: 10.1371/journal.pgph.0006698 (PMC13353989; doi:10.1371/journal.pgph.0006698)
Supplement: Attachment — Submitted filename: Senegal_debt_healthspending_PGPH_responserevs_final_5.docx [file pgph.0006698.s005.docx]

**Response to reviewers: ”Influence of Public External Debt on Government Health Spending: A Mixed-Methods Case Study of Senegal”**

First of all, the authors would like to thank the reviewers for their time and effort in improving the quality of our manuscript. As outlined below, we have made a series of revisions following reviewer comments, which have significantly strengthened the paper. Where we have kept the manuscript as originally submitted, detailed rationales for doing so have been provided.

We hope for a positive reception of this resubmission.

**Comments to the Author**

Reviewer #1: The paper is well written, and I recommend publishing it. The study clearly showed the current dynamics of the debt influence on the government health spending in particular and the economy in general.

The authors would like to thank the reviewer for this immediately positive reception.

Reviewer #2: The authors have chosen a highly promising area of study that makes a valuable contribution to health policy dialogue. The analysis is comprehensive, integrating both quantitative and qualitative data. The topic is highly relevant and timely, addressing a critical issue in the health financing systems of low- and middle-income countries. Despite the study's strengths, several important gaps remain before it can be considered for publication, as detailed in the manuscript.
In the title, the authors should provide a clear rationale for using "health spending" instead of "health expenditure." In the introduction, for example, comparing Senegal’s performance with other African countries using the Abuja Declaration as a benchmark would help contextualize Senegal’s case. In the methods section, the framework needs revision to establish its foundation in theory, clearly showing the position of external assistance for health/donors, while also strengthening statistical rigor in explaining the relationship between external debt and government health spending.
The results section may benefit from incorporating the document analysis approach claimed in the methods section. The discussion section would be stronger if it clearly addressed the direct, indirect, and political decision-making effects, following the flow of the results. The conclusion section should also be revised, starting with the section title, and should include "recommendations."

We thank the reviewer for their positive description of the study’s strengths. The described study limitations are addressed below in separate comments.

**Attached word-file: “Debt comment”:**

Influence of Public External Debt on Government Health Spending: A Mixed-Methods Case Study of Senegal reflects the real-world external debt situation in the developing countries. So it is well done and data results are well documented. I recommend to be published and displayed on websites. Accepted for publication.

Again, thank you very much for this positive reception.

**Attached annotated PDF-file: “5PGPH-D-25-03785 “**

First of all, we thank the reviewer for having provided clear and thorough comments, which we have implemented to the best of our ability and as we have deemed most appropriate. The comments from the PDF file are copied below, while **the provided page and line numbers refer to the submitted revised manuscript in word format (without tracked changes).**

- Title:
- The authors should provide a clear rationale for using "health spending" instead of "health expenditure."

We have changed to health expenditure as per above.

P. 1, l. 1: Why " Health spending preferred instead of Health expenditure". Spending is broader more general ; is that what the authors want to see? Or the actual health disbursement more explained as " health expenditure". The study also looks in government health expenditure at the end.

Good point, thank you, this has now been changed to health expenditure.

P. 2, l. 6: The " mixed methods case study" should be repositioned under the method section, with the current sentence rewritten to preserve its intended meaning.

Great observation, thank you, this has now been changed as follows:

“We aimed to better understand these relationships through a case study of Senegal.

**Methods**

“We conducted a mixed-methods case study using descriptive quantitative analyses of loan, debt and health financing statistics in Senegal from 1970-2023. We also used the Framework method to thematically analyse semi-structured interview data collected between October 2019 and January 2020 from 25 key informants from government ministries, development partners, academia, civil society and hospital management in Senegal. We triangulated these data with findings from a purposive document review of reports from government websites and academic literature discussing loan and debt effects on government health expenditure in Senegal.”

P. 2, l. 9: Lacks brief sentences of data analysis method

Good point, now added as per above:

“We conducted a mixed-methods case study using descriptive quantitative analyses of loan, debt and health financing statistics in Senegal from 1970-2023. We also used the Framework method to thematically analyse semi-structured interview data collected between October 2019 and January 2020 from 25 key informants from government ministries, development partners, academia, civil society and hospital management in Senegal. We triangulated these data with findings from a purposive document review of reports from government websites and academic literature discussing loan and debt effects on government health expenditure in Senegal.”

P. 3, l. 24: “This section reads more like recommendations though it labelled as a conclusion. It should begin with brief concluding statement and then transition smoothly in to the current recommendations.”

Thank you, very valid point. We have now added the below sentence and continue with our recommendations:

“Overall, our findings indicate that Senegal’s debt burden has constrained government health expenditure, serving as a possible co-determinant of the regression seen on the Abuja pledge since 2000. To mitigate the negative effect of debt repayment, official external creditors should consider more flexible debt repayment timelines and exploring options for debt relief including debt-to-health swaps. Supported by debt relief, increased government priority of the health sector working toward the Abuja pledge will be necessary to make progress on universal health coverage. We encourage further country level research documenting debt impacts on health financing.”

*The following four comments all relate to the same issue, which we have addressed jointly below.*

- Methods:
- In the methods section, the framework needs revision to establish its foundation in theory, clearly showing the position of external assistance for health/donors, while also strengthening statistical rigor in explaining the relationship between external debt and government health spending.

P. 5, l. 2: “While developing the potential path ways is valuable contribution, it must be supported by previously established clear theoretical foundation.”

P. 5, l. 2 (regarding development assistance): “The frame work doesn't indicate, this while it remains one determinant of government health spending.”

P. 5, l. 2: “It is important to introduce the readers with " what public external debt is and how/mechanism through which it could affect public health spending" before resonating its burden.”

Thank you, these are valid critiques. After much consideration, we have opted to remove the framework figure and the corresponding section from the methods section, because it basically just displayed some simple fiscal considerations under budget constraint, without a deep foundation in economic theory. This has allowed us to move a condensed description of these basic theoretical concepts to where the reviewer has requested them: in the very beginning of the introduction. In doing so, we have strengthened the theoretical foundation by providing relevant referencing. The paragraph now reads as follows:

“Across Low- and Middle-Income Countries (LMICs), external official creditors provide loans to recipient governments as general budget support or earmarked funds to specific sectors, including the health sector. From the perspective of the health sector in a given country, loans may thereby help grow the health budget directly through lending for health, or indirectly through loans stimulating economic growth and government revenue raising, which can in turn be allocated for health purposes. Over time however, debt plus its interest accumulates and needs repayment from the general government budget, which can affect the fiscal space available for sectoral budget allocations - including for health (1-5). This results in a question of relative balance: Which sectors benefit more from development lending, and which sectors are “picking up the tab” for repaying investments in other sectors?”

As for a description of the role of donors and aid, as previously mentioned, this is partly due to our findings pertaining to donors and associated theoretical considerations having been previously published in Health Policy and Planning: <https://doi.org/10.1093/heapol/czae110>

However, the present paper does indeed discuss the role of donors and aid as these relate to debt and health financing. We have now emphasized this part of this discussion further, in particular by drawing in our finding of possible negative synergy between aid and debt:

“Additionally, it deserves mention that while this paper focused on the modality of development lending, we acknowledge the important EDP contribution of providing grant financing. In spite of its decreasing share over time, it has provided 81% of ODA for the health sector in Senegal on average between 2002-2023 [50]. The significant overall ODA funding flows to the health sector in Senegal offer another explanation for why this sector has not been prioritised by the Senegalese government through aid displacement of government funds (fungibility, discussed for Senegal in another article [77]) [28, 30, 77-90]. This has the same negative direction of effect on health financing as the identified debt constraint, constituting a dual challenge for revitalising health spending in Senegal, and potentially in other countries experiencing similar fiscal conditions. Importantly, a donor pointed to a possible point of negative synergy, whereby they deemed the health budget more likely to be cut in order to meet debt obligations because of the presence of donors to cover costs. While not brought up by other interviewees, this observation warrants further exploration both in Senegal and elsewhere, as a potentially detrimental interaction between these two external macroeconomic factors.

Finally, the recent cuts to ODA from the United States, France, Germany and other donors will leave a funding shortfall across sectors in Senegal [91, 92]. To mitigate this and avoid adverse consequences for the Senegalese people, other partners and/or the Senegalese government will have to step in and cover costs. The latter adds the risk of necessitating external borrowing, which could further fuel downstream debt constraints on the health sector, in turn strengthening the case for debt relief in Senegal (see Conclusions section).”

P. 5, l. 12: The term " high" should better be expressed in a more explicit way

Thank you, good point, we have now made this clearer as follows:

“In the late 1990’s and early 2000’s, the public external debt burdens of Low- and Middle-Income Countries (LMICs) were relatively high: average PPG external debt stocks hovered around 20% of Gross Domestic Product (GDP) and PPG external debt servicing levels around 2.5% of GDP, with great variation between countries, regions and income groups (Figure 2).”

P. 5, l. 25: It is recommended to link this calls also with the SDG or elsewhere in the introduction to provide stronger contextual ground.

We appreciate this invitation to strengthen the case for our research even further. The sentence in question now reads:

“Recent calls have been made for debt resolution, including by the United Nations and the WB, to ensure funding can be sustained for public sectors, including health sectors, and the Sustainable Development Goals (SDGs) in general (e.g. [11-20]).”

P. 5, l. 32: Add few more country specific studies by region is recommended to further strengthen the evidences.

Thank you. Through extensive literature review conducted for a PhD thesis, we have only identified the two studies from Kenya mentioned in the manuscript. We have now made this clearer in the text:

“We were only able to identify two single-country case studies, both from Kenya, that used respectively mixed and quantitative methods to examine links between the country’s debt burden and government health spending [29, 30].”

P. 5, l. 35: Link this as a challenge for the path to UHC.

Great suggestion, we have done so as follows:

“The majority of the identified studies find negative associations between debt burdens and government health spending. This is similar to most of the identified econometric literature on public debt and education spending, mostly finding a negative link [21, 22, 33-39]. For health, this indicates a challenge for the path to Universal Health Coverage (UHC) (42).”

- Introduction -
- As an example, comparing Senegal’s performance with other African countries using the Abuja Declaration as a benchmark would help contextualize Senegal’s case.

Great suggestion, thank you, we have now added the following paragraph to the introduction (this also addressed a corresponding in-text comment in the PDF file on the same matter):

P. 6, l. 17: “At 3.3%, its level of domestic government health spending (Government Health Spending as a Source, GHE-S) out of General Government Expenditure (GGE) also ranked as number 44 out of 49 SSA countries with data in 2022 [9]. Even when including external transfers distributed by the government (on-budget development assistance for the health sector), bringing government health expenditure to 4.9% of GGE, this is still far from the pledge made by African Union countries in 2001 as part of the Abuja declaration to spend 15% of government budgets on the health sector [9, 38].”

*The following two comments raise similar issues and are therefore addressed jointly below.*

P. 7, l. 14: “In this section; sampling and sample selection technique ( for the qualitative), for the quantitative; variables ( explanatory and outcome), variable definition and data sources was not clearly presented. Given their importance, the investigators should clearly include these details.

Also, the section doesn't address the quantitative and the qualitative separately. Hence, it needs to be presented with greater clarity”

P. 7, l. 14: “This section requires revision to clearly and explicitly explain the two approaches (quantitative and qualitative) separately and in-detail”

Thank you very much for these comments. Regarding qualitative sampling, this has already been addressed in response to a related reviewer comment for p. 8, l. 25. As for variable descriptions and definitions, this relates to the fact that our paper is not econometric. We have provided a detailed rationale for this in response to another reviewer comment regarding this matter for p. 19, l. 27. Regarding data sources, we have now provided additional detail as follows (also provided in response to a previous comment for p. 7, l. 26):

P. 7, l. 27: “We assembled publicly available secondary data on PPG loan disbursements in Senegal separated by creditor type and PPG external debt stock and service from the WB Open Data and International Debt Statistics databases (8, 12); general government revenue and expenditure data from the IMF Public Finances in Modern History and WB Open Data databases (8, 49); total official development loans received by sector from the OECD Creditor Reporting System (50); and GHE-S, External health financing (EXT), OOP and Voluntary Health Insurance (VHI) from the World Health Organization (WHO) Global Health Expenditure Database (14).”

In regard to the study approach section, this is intentionally written to provide a summary overview of the study methods used. The following three sections then provide the details of each separate method, grouped separately under quantitative and qualitative methods as appropriate. In the authors’ opinion, a mixed methods paper should have an introductory methods paragraph that provides an overview of the study approach, before delving into the details of each method used. Dividing the introductory study approach section would also entail repetition from the sections that follow, where the details of each method are provided.

Rather than dividing our study approach section into separate parts, we have instead provided better signposting to make our structure clearer to the reader:

P. 7, l. 25 (the end of the study approach section): “The following sections describe our three different methods applied.”

Methods section subheadings:

P. 7, l. 26: “Quantitative data collection and analysis”

P. 8, l. 25: “Qualitative data collection and analysis”

*…*

*Interviews*

*…*

*Document review”*

We kindly hope for the reviewer’s understanding of this decision.

P. 7, l. 26: “Before describing how the data is analyzed, it is important to first explain how the data was collected including the data collection method, instrument by which the data was collected and how the tool was validated”

Thank you very much for this comment. We did not collect any primary data using any instruments or tools. We instead assembled secondary data from publicly available online databases. We have now made this clarification in the methods section as follows (p. 7, l. 26):

### “Quantitative data collection and analysis

We assembled publicly available secondary data on PPG loan disbursements in Senegal separated by creditor type and PPG external debt stock and service from the WB Open Data and International Debt Statistics databases (8, 12). General government revenue and expenditure data were obtained from the IMF Public Finances in Modern History and WB Open Data databases (8, 50); total official development loans received by sector from the OECD Creditor Reporting System (51); and GHE-S, External health financing (EXT), OOP and Voluntary Health Insurance (VHI) from the World Health Organization (WHO) Global Health Expenditure Database (14).”

P. 8, l. 25: “This section looks intermixed, the description of sampling, sample size, sampling technique, needs to be clearly described. The section also needs to be subtitled something like" qualitative data collection and analysis.." that acomodates the section message”

Thank you very much for this comment. We have now strengthened this section by adding the requested details. The section now reads as follows (p. 8, l. 25):

“Qualitative data collection and analysis

*Interviews*

To identify potential key informants, we first purposively mapped stakeholders in Senegal with roles relevant to health financing and macro-fiscal policy based on author knowledge and searching relevant organisation websites. We then began contacting identified individuals and initiated interviews, and then used snowball sampling to identify further key informants. A total of 32 key informants were identified for interview, of which 25 agreed to participate. Our final sample included representatives of four bilateral development partners, four multilateral development partners, 9 from central government across four different ministries, four from Senegalese civil society organisations, three from academia and one from hospital management.

Interviews were conducted in Dakar between September 30, 2019, to January 31, 2020, by FF, who was a PhD student at the time. He had completed a course on SSIs at LSHTM before conducting interviews. He was supervised by MMA, who had extensive experience with SSIs before this study. We used the Semi-Structured Interview (SSI) method, allowing the interviewer to probe and explore interviewee responses in further details as relevant. Our interview topic guides were tested through conducting preliminary mock interviews with an external researcher, who provided critical feedback on both content and interview style, which was integrated before commencing stakeholder interviews. Interviews were conducted in French or English, using an interpreter for interviews in French. Interviews were recorded and transcribed by a professional transcriptionist, where consent was given for this. The participant information sheet used, consent form and an example interview topic guide can be found in the online LSHTM data repository (51) and in Appendix 1.

The interview data were analysed using the Framework method (52). Themes were established deductively based on the research questions as well as inductively from the interviews. The thematic coding framework was validated by an independent researcher before coding all interview data, using NVivo 12 (53). Participant views and key quotes were summarised across themes in a Framework matrix (52, 54). Identified patterns in these summary findings were then integrated into the results section. All interviewee statements were anonymised when writing up the paper.”

The section then continues by describing our other qualitative method: document review.

P. 9, l. 4: “This section doesn't tell about who data collectors are, supervisors, training given, how the tool was validated...”

Thank you very much for this comment. We have now added the requested details as per below:

“We used the Semi-Structured Interview (SSI) method, allowing the interviewer to probe and explore interviewee responses in further details as relevant. Interviews were conducted by FF, who was a PhD student at the time. He had completed a course on SSIs at LSHTM before conducting interviews. He was supervised by MMA, who had extensive experience with SSIs before this study. Our interview topic guide was tested through conducting a preliminary mock interview with an external researcher, who provided critical feedback on both content and interview style, which was integrated before commencing stakeholder interviews.”

P. 9, l. 21: “Why these documents were chosen? Needs justification.”

This relates to the choice of IMF/WB documents for review. We thank the reviewer for bringing this up, and we have now added the following justification to the methods section:

“We searched Senegalese government websites [55-60], and the Senegalese websites of the International Monetary Fund (IMF) [61, 62] and the World Bank (WB) [63] for documents discussing an influence of public external lending on government health spending (215 full text documents reviewed). We chose to review documents from the IMF and WB due to the central, privileged positions of these institutions in monitoring, analysing and advising on fiscal policy, as summarised in numerous staff reports, strategy papers and more.”

P. 9, l. 33: who reviewed, How?

Thank you, we have now added information on this as per below:

“The document review was carried out by FF, who read through the documents while applying our inclusion criteria and integrating relevant findings into the results section in stages from the time interviews were conducted in 2019/20 up until March 2025.”

P. 10, l. 8: “This section mixes trend description with interpritation. It would be better to begin with a straightforward introduction to the results, using a short linking sentence that connects to the second paragraph.”

Thank you very much, good point. We have rewritten this introductory paragraph as follows:

P. 10, l. 8: “In this section, we analyse Senegal’s borrowing and debt stock and servicing levels over time in relation to GDP and government expenditure, including on health”

P. 11, l. 16: If this part is intended as a foot note, it is too long; condensing is necessary.

Indeed, we have now shortened this caption as per below and moved the full caption to the appendix for reference.

“Figure 2: **Panel A**: Public and Publicly Guaranteed (PPG) borrowing in Senegal separated by creditor type, 1970-2023 (billion constant 2023 US$) [3, 7]. **Panel B**: PPG debt stock in Senegal from 1970-2023 [7]. **Panel C**: Gross Domestic Product (GDP) (1970-2023), PPG external debt stock (1970-2023), and General Government Expenditure (GGE) in Senegal (1994-2022) [3, 49]. **Panel D**: PPG external debt service (1970-2023) and Government Health Expenditure as a Source (GHE-S) (2000-2022) in Senegal [3, 9]. Please see Appendix 1 for additional details on the numbers used to produce this figure.”

- Results:
- The results section may benefit from incorporating the document analysis approach claimed in the methods section.

Thank you for this comment. The document analysis was integrated in the originally submitted version. We have copied an example below for your reference:

P. 15, l. 7: “A constraining effect of debt on social spending in Senegal has been acknowledged in official country reports both when debt levels were highest around the turn of the millennium, and again in 2020. The 1998 Enhanced Structural Adjustment Facility policy framework paper recognised that “*the fiscal burden* [of external debt] *remains high, however, with debt service to revenues projected to be 38 percent in 1998. This makes it more difficult to increase expenditures, including for the priority sectors of education and health”* (68). In the 2002 Poverty Reduction Strategy Paper (PRSP), it was similarly stated that: “*the country’s debt burden is a major obstacle to efficient allocation of public resources in favour of the social sectors*” (69). In the recent context of Senegal’s growing debt burden, a 2020 IMF country report for Senegal stated that: *“… debt service continues to absorb a sizable portion of fiscal revenues, thus limiting room for other expenditures in critical areas such as infrastructure investment, health, and education”* (70).”

*The following two comments raise similar issues in the provided summary comments and detailed PDF-comments respectively and are therefore addressed jointly below.*

- Discussion and conclusion:
- The discussion section would be stronger if it clearly addressed the direct, indirect, and political decision-making effects, following the flow of the results.

P. 16, l. 22: “The discussion section will benefit , if it visibly discuss the finding that claiming that debt constrained the health budget; in terms of direct effect- say the debt obligation crowd out fiscal space. The indirect effect say higher lending to the economic sector doesn't translated to higher health sector spending, in-fact the findings points to opposite, it is declining. Also the political decision effect; health is not prioritized relative to say education -though education as social determinant of health is expected to improve health.”

Thank you very much for this comment. In terms of the subject matter, the requested discussion points are already in the paper (not copied here for conciseness). The direct debt finding is discussed on p. 16, l. 30- p. 18, l. 6. The absence of an indirect effect is discussed on p. 16, l. 33 - p. 18, l. 15, however the finding could have been more clearly explained. We have therefore edited our reiteration of this finding in the beginning of the discussion section as follows:

P. 16, l. 25: “While development lending has supported the economic growth and government revenue and expenditure expansion seen since 2000, as well as directly supporting the health sector, any indirect economic effects have been largely cancelled out by decreasing relative budget prioritization of the health sector.”

The political prioritization dimension is discussed on p. 17, l. 10-p. 18, l. 24.

In terms of structure, our discussion section is structured according to importance of findings. Our main hypothesis and associated finding was that of a debt effect on health expenditure, which we discuss first. The other dimensions then follow this main finding, in a hierarchy of importance. Additionally, the current flow of the discussion (which has followed deep reflection, discussion and editing before submission) has the strength of weaving together the different financial direct and indirect and political effects, all the way from loans to both health and other sectors onto debt repayments and health expenditure implications. In the authors’ opinion, this elevates the discussion to a point of synergy, seeking to leave the reader with a more comprehensive and coherent understanding of how these different effects combine - which is very much in the spirit of the mixed methods approach. We feel that restructuring the discussion section into the different parts as suggested would remove this synergy and associated higher-level analytical comprehension and interpretation of the different study hypotheses and findings, as well as going against the very commonly applied structuring by hierarchy of importance.

We kindly hope for the reviewer’s understanding of this decision.

*The following two comments raise similar issues and are therefore addressed jointly below.*

P. 16, l. 35: This should be emphasized and discussed in discussion section in comparison with the Abuja declaration.

P. 16, l. 35: This section would be stronger if it explicitely linked to relevant policy framework like the Abuja declaration bench mark. What the current findings looks like in comparison with that of 15% pledged, comparable? below?...Although the Abuja declaration is mentioned later in the findings, its visibility and integration are not as they should be.

This is a great point, and we agree that further emphasis should be placed on this key finding. We have now done so in the discussion section, reiterating this finding and stressing its importance in relation to the Abuja pledge and thereby to UHC and the other health SDGs:

“GHE-S however decreased as a share of GGE (from 9.0% in 2000 to 3.4% in 2022 (Figure 5) [3, 9]), and it did not grow in real, absolute terms between 2006 and 2022 (using US$ GDP deflator [3]). This decoupling deviates from findings across 133 LMICs, showing that around 70% of the budgetary space for health is driven by changes in overall government expenditure, with the remaining 30% being attributable to the expenditure budget share allocation to the health sector [71]. In Senegal, the health sector’s share allocation has decreased so much that any effects from growth in GDP, revenue and expenditure have been largely cancelled out. Undoing this continuous regression on the Abuja pledge of 15% of the government budget being allocated to the health sector will be vital for making sustained progress on UHC and other health SDGs in Senegal.”

P. 18, l. 25: The investigators should consider the role of donor if as a factor it has negative positively affected healthcare spending in view of the debt effect - this was not visible in the expected sections; framework, result, discussion. How ever donor support has key role directly and indirectly.

Thank you very much for this valid comment. This is partly due to our findings pertaining to donors having been previously published in Health Policy and Planning: <https://doi.org/10.1093/heapol/czae110>

However, the present paper does discuss the role of donors and aid as these relate to debt and health financing. We have now emphasized this part of this discussion further, in particular by drawing in our finding of possible negative synergy between aid and debt:

“Additionally, it deserves mention that while this paper focused on the modality of development lending, we acknowledge the important EDP contribution of providing grant financing. In spite of its decreasing share over time, it has provided 81% of ODA for the health sector in Senegal on average between 2002-2023 [50]. The significant overall ODA funding flows to the health sector in Senegal offer another explanation for why this sector has not been prioritised by the Senegalese government through aid displacement of government funds (fungibility, discussed for Senegal in another article [77]) [28, 30, 77-90]. This has the same negative direction of effect on health financing as the identified debt constraint, constituting a dual challenge for revitalising health spending in Senegal, and potentially in other countries experiencing similar fiscal conditions. Importantly, a donor pointed to a possible point of negative synergy, whereby they deemed the health budget more likely to be cut in order to meet debt obligations because of the presence of donors to cover costs. While not brought up by other interviewees, this observation warrants further exploration both in Senegal and elsewhere, as a potentially detrimental interaction between these two external macroeconomic factors.

Finally, the recent cuts to ODA from the United States, France, Germany and other donors will leave a funding shortfall across sectors in Senegal [91, 92]. To mitigate this and avoid adverse consequences for the Senegalese people, other partners and/or the Senegalese government will have to step in and cover costs. The latter adds the risk of necessitating external borrowing, which could further fuel downstream debt constraints on the health sector, in turn strengthening the case for debt relief in Senegal (see Conclusions section).”

*The following two comments raise similar issues and are therefore addressed jointly below.*

P. 19, l. 27: “The analysis could benefited from a formal econometric analysis as the current descriptive analysis cannot quantify the strength of the relationship the investigators claim in B tough they approached qualitatively.”

P. 19, l. 27: “This section needs greater attention to strengthen the quantitative part of the research particularly in detailing the model used, its specifications and estimation methods, by referring to relevant econometric literatures”

Thank you for these comments. They have encouraged both reflection and exploratory modelling. We originally opted not to conduct a formal econometric analysis for this paper for a number of reasons as outlined below. Upon receiving this comment, we have now performed some exploratory modelling, which has confirmed our original decision. Here is a summary of our reasoning:

a. The focus, main strength and novelty of this paper, in our opinion, lies very much in its original data collection through interviews with centrally placed stakeholders in Senegal, providing key insights into the political economy of Senegal as it relates to health financing. The document review and quantitative analysis of secondary data supplement and triangulate our interview findings, and the three methods combined provide a comprehensive exploration into our research question in and of themselves.
b. In response to the reviewer comments, we have run univariate ARDL and first-differenced OLS models for our time-series data on GHE-S, PPG external debt stock and debt service variables. These simple regressions do not show a significant relationship between debt variables and GHE-S. However, as soon as you start introducing covariates to adjust for confounding such as GDP, a Covid-dummy, population, etc., these findings change substantially and we start seeing some significant and borderline significant negative relationships between debt variables and GHE-S. A simple univariate regression analysis thereby risks committing a type II error mainly through not adjusting for confounding. The size and significance of the relationships of interest is however dependent on model specification.
c. A properly conducted formal econometric analysis would require the following considerations, among others:
   - Which covariates to include: GDP, population, IMR/U5MR/dependency ratio, ODA for health and non-health purposes, on-budget and off-budget, etc. This in itself would necessitate a comprehensive literature review to theoretically justify a given model specification.
   - With a limited time series such as ours, the degrees of freedom are quickly exhausted once theoretically justified controls (GDP, population, a Covid-19 shock, etc.) are introduced. Any resulting significance would therefore be highly susceptible to overfitting.
   - How to specify variables – absolute, per capita, per gdp, log-transformed or not, etc; Choice of lag structure – 1 year, 2 years, none?; Considering interaction terms.
   - Diagnostic testing for non-stationarity, cointegration, multicollinearity, etc.
   - Choice of best estimator(s)
   - Sensitivity analysis: Given the number of considerations outlined above, extensive sensitivity testing would be necessary for an econometrically sound analysis.

This could be a potential avenue for future research and possibly a stand-alone paper, however it falls beyond the scope of the present paper which has a different methodological focus. Importantly, it would likely suffer from methodological issues mainly through type II error and omitted variable bias in the case of a short model, or overfitting in the case of a more comprehensive model.

We have explored the relationships between GHE-S and debt variables across a panel of more than 105 countries in another paper (forthcoming in Health Policy and Planning), where the larger panel allowed us to conduct a nuanced exploration of the relationships in question.

We have now added the following paragraph to the limitations section:

P. 19, l. 27: “We explored the feasibility of a formal econometric analysis, however, the short time series with limited degrees of freedom rendered a theoretically justified multiple regression model infeasible due to limited power and risk of overfitting, while a more parsimonious specification risked omitted variable bias and associated erroneous statistical inference.”

We kindly hope the reviewer will appreciate this rationale.

*The following two comments raise similar issues in the provided summary comments and detailed PDF-comments respectively and are therefore addressed jointly below.*

“The conclusion section should also be revised, starting with the section title, and should include "recommendations."

P. 20, l. 12: “This section title should be rewritten as " Conclusion and Recommendation" for more clarity; The conclusion needs to be explained separately, making concise yet comprehensive, summarizing the key insights of the study.

The Recommendation subsection should explicitly outline recommendation that inform stakeholders such as policymakers, planners, and other relevant actors in-line with the findings from the research.”

Thank you, we have now changed the title to “Conclusions and recommendations”.

We have also added the following paragraph in the beginning of the conclusion section to reiterate the main study conclusions:

P. 20, l. 13: “Our findings indicate that Senegal’s debt has constrained the general government expenditure budget and thereby the health sector budget. While development lending has supported economic growth and government revenue and expenditure expansion, as well as directly supporting the health sector, any indirect economic effects have been largely cancelled out by decreasing relative budget prioritization of the health sector. Unexpectedly, the education sector appeared to have been insulated from debt effects due to a higher political prioritisation.”

The structure of the conclusion section now follows:

- Main study conclusions
- Novelty of these,
- Policy recommendations
- Research recommendations

As for policy recommendations, these are provided in the original submission, p. 20, l. 27- p. 21, l. 19, making specific mention of which stakeholders they are intended for (Government of Senegal, external development partners, IMF, and advocates) (not copied here for conciseness).
