# Supplementary material for: Influence of public external debt on government health expenditure: A mixed-methods case study of Senegal
Source: PLOS Glob Public Health. 2026 Jul 10;6(7):e0006698. doi: 10.1371/journal.pgph.0006698 (PMC13353989; doi:10.1371/journal.pgph.0006698)
Supplement: Peer review history

# Peer review: Decision Letter 0

**DOI:** 10.1371/journal.pgph.0006698.r001

**Authors:** Damen Haile Mariam

11 Feb 2026

PGPH-D-25-03785

Influence of Public External Debt on Government Health Spending: A Mixed-Methods Case Study of Senegal

PLOS Global Public Health

Dear Dr. Federspiel,

Thank you for submitting your manuscript to PLOS Global Public Health. After careful consideration, we feel that it has merit but does not fully meet PLOS Global Public Health’s publication criteria as it currently stands. Therefore, we invite you to submit a revised version of the manuscript that addresses the points raised during the review process.

Please submit your revised manuscript by Mar 28 2026 11:59PM. If you will need more time than this to complete your revisions, please reply to this message or contact the journal office at globalpubhealth@plos.org. When you're ready to submit your revision, log on to https://www.editorialmanager.com/pgph/ and select the 'Submissions Needing Revision' folder to locate your manuscript file.

Please include the following items when submitting your revised manuscript:

- A letter that responds to each point raised by the editor and reviewer(s). You should upload this letter as a separate file labeled 'Response to Reviewers'.
- A marked-up copy of your manuscript that highlights changes made to the original version. You should upload this as a separate file labeled 'Revised Manuscript with Track Changes'.
- An unmarked version of your revised paper without tracked changes. You should upload this as a separate file labeled 'Manuscript'.

Guidelines for resubmitting your figure files are available below the reviewer comments at the end of this letter.

We look forward to receiving your revised manuscript.

Kind regards,

Damen Haile Mariam, MD, MPH, PhD

Academic Editor

PLOS Global Public Health

Journal Requirements:

1. Please include a complete copy of PLOS’ questionnaire on inclusivity in global research in your revised manuscript. Our policy for research in this area aims to improve transparency in the reporting of research performed outside of researchers’ own country or community. The policy applies to researchers who have travelled to a different country to conduct research, research with Indigenous populations or their lands, and research on cultural artefacts. The questionnaire can also be requested at the journal’s discretion for any other submissions, even if these conditions are not met. Please find more information on the policy and a link to download a blank copy of the questionnaire here: https://journals.plos.org/globalpublichealth/s/best-practices-in-research-reporting. Please upload a completed version of your questionnaire as Supporting Information when you resubmit your manuscript.

2. Please provide separate figure files in .tif or .eps format.

For more information about figure files please see our guidelines:  LINK

https://journals.plos.org/globalpublichealth/s/figures

https://journals.plos.org/globalpublichealth/s/figures#loc-file-requirements

If the reviewer comments include a recommendation to cite specific previously published works, please review and evaluate these publications to determine whether they are relevant and should be cited. There is no requirement to cite these works unless the editor has indicated otherwise.

Additional Editor Comments (if provided):

Reviewer 1 -

- Title:

- The authors should provide a clear rationale for using "health spending" instead of "health expenditure."

- Introduction -

- As an example, comparing Senegal’s performance with other African countries using the Abuja Declaration as a benchmark would help contextualize Senegal’s case.

- Methods:

- In the methods section, the framework needs revision to establish its foundation in theory, clearly showing the position of external assistance for health/donors, while also strengthening statistical rigor in explaining the relationship

between external debt and government health spending.

- Results:

- The results section may benefit from incorporating the document analysis approach claimed in the methods section.

- Discussion and conclusion:

- The discussion section would be stronger if it clearly addressed the direct, indirect, and political decision-making effects, following the flow of the results.

The conclusion section should also be revised, starting with the section title, and should include "recommendations."

[Note: HTML markup is below. Please do not edit.]

Reviewers' comments:

Reviewer's Responses to Questions

**Comments to the Author**

1. Does this manuscript meet PLOS Global Public Health’s publication criteria? Is the manuscript technically sound, and do the data support the conclusions? The manuscript must describe methodologically and ethically rigorous research with conclusions that are appropriately drawn based on the data presented.

Reviewer #1: Yes

Reviewer #2: Partly

**********

2. Has the statistical analysis been performed appropriately and rigorously?

Reviewer #1: Yes

Reviewer #2: Yes

**********

3. Have the authors made all data underlying the findings in their manuscript fully available (please refer to the Data Availability Statement at the start of the manuscript PDF file)?

The PLOS Data policy requires authors to make all data underlying the findings described in their manuscript fully available without restriction, with rare exception. The data should be provided as part of the manuscript or its supporting information, or deposited to a public repository. For example, in addition to summary statistics, the data points behind means, medians and variance measures should be available. If there are restrictions on publicly sharing data—e.g. participant privacy or use of data from a third party—those must be specified.

Reviewer #1: Yes

Reviewer #2: Yes

**********

4. Is the manuscript presented in an intelligible fashion and written in standard English?

PLOS Global Public Health does not copyedit accepted manuscripts, so the language in submitted articles must be clear, correct, and unambiguous. Any typographical or grammatical errors should be corrected at revision, so please note any specific errors here.

Reviewer #1: Yes

Reviewer #2: Yes

**********

5. Review Comments to the Author

Please use the space provided to explain your answers to the questions above. You may also include additional comments for the author, including concerns about dual publication, research ethics, or publication ethics. (Please upload your review as an attachment if it exceeds 20,000 characters)

Reviewer #1: The paper is well written, and I recommend publishing it. The study clearly showed the current dynamics of the debt influence on the government health spending in particular and the economy in general.

Reviewer #2: The authors have chosen a highly promising area of study that makes a valuable contribution to health policy dialogue. The analysis is comprehensive, integrating both quantitative and qualitative data. The topic is highly relevant and timely, addressing a critical issue in the health financing systems of low- and middle-income countries. Despite the study's strengths, several important gaps remain before it can be considered for publication, as detailed in the manuscript.

In the title, the authors should provide a clear rationale for using "health spending" instead of "health expenditure." In the introduction, for example, comparing Senegal’s performance with other African countries using the Abuja Declaration as a benchmark would help contextualize Senegal’s case. In the methods section, the framework needs revision to establish its foundation in theory, clearly showing the position of external assistance for health/donors, while also strengthening statistical rigor in explaining the relationship between external debt and government health spending.

The results section may benefit from incorporating the document analysis approach claimed in the methods section. The discussion section would be stronger if it clearly addressed the direct, indirect, and political decision-making effects, following the flow of the results. The conclusion section should also be revised, starting with the section title, and should include "recommendations."

**********

6. PLOS authors have the option to publish the peer review history of their article (what does this mean?). If published, this will include your full peer review and any attached files.

**Do you want your identity to be public for this peer review?**  If you choose “no”, your identity will remain anonymous but your review may still be made public.

For information about this choice, including consent withdrawal, please see our Privacy Policy.

Reviewer #1: **Yes:**  Dr Meseret Molla Kassahun

Reviewer #2: No

**********

[NOTE: If reviewer comments were submitted as an attachment file, they will be attached to this email and accessible via the submission site. Please log into your account, locate the manuscript record, and check for the action link "View Attachments". If this link does not appear, there are no attachment files.]

-->Figure Resubmissions:

While revising your submission, we strongly recommend that you use PLOS’s NAAS tool (https://ngplosjournals.pagemajik.ai/artanalysis) to test your figure files. NAAS can convert your figure files to the TIFF file type and meet basic requirements (such as print size, resolution), or provide you with a report on issues that do not meet our requirements and that NAAS cannot fix.

After uploading your figures to PLOS’s NAAS tool - https://ngplosjournals.pagemajik.ai/artanalysis, NAAS will process the files provided and display the results in the "Uploaded Files" section of the page as the processing is complete. If the uploaded figures meet our requirements (or NAAS is able to fix the files to meet our requirements), the figure will be marked as "fixed" above. If NAAS is unable to fix the files, a red "failed" label will appear above. When NAAS has confirmed that the figure files meet our requirements, please download the file via the download option, and include these NAAS processed figure files when submitting your revised manuscript.

Attachment Submitted filename: debt comment.docx

Attachment Submitted filename: 5PGPH-D-25-03785.pdf

# Author comment: Author response to Decision Letter 1

**DOI:** 10.1371/journal.pgph.0006698.r002

21 May 2026

Attachment Submitted filename: Senegal_debt_healthspending_PGPH_responserevs_final_5.docx

# Editor report: Decision Letter 1

**DOI:** 10.1371/journal.pgph.0006698.r003

**Authors:** Damen Haile Mariam, Damen Haile Mariam

2 Jun 2026

Influence of Public External Debt on Government Health Expenditure: A Mixed-Methods Case Study of Senegal

PGPH-D-25-03785R1

Dear Dr. Federspiel,

We are pleased to inform you that your manuscript 'Influence of Public External Debt on Government Health Expenditure: A Mixed-Methods Case Study of Senegal' has been provisionally accepted for publication in PLOS Global Public Health.

Before your manuscript can be formally accepted you will need to complete some formatting changes, which you will receive in a follow up email. A member of our team will be in touch with a set of requests.

Please note that your manuscript will not be scheduled for publication until you have made the required changes, so a swift response is appreciated.

IMPORTANT: The editorial review process is now complete. PLOS will only permit corrections to spelling, formatting or significant scientific errors from this point onwards. Requests for major changes, or any which affect the scientific understanding of your work, will cause delays to the publication date of your manuscript.

If your institution or institutions have a press office, please notify them about your upcoming paper to help maximize its impact. If they'll be preparing press materials, please inform our press team as soon as possible -- no later than 48 hours after receiving the formal acceptance. Your manuscript will remain under strict press embargo until 2 pm Eastern Time on the date of publication. For more information, please contact globalpubhealth@plos.org.

Thank you again for supporting Open Access publishing; we are looking forward to publishing your work in PLOS Global Public Health.

Best regards,

Damen Haile Mariam, MD, MPH, PhD

Academic Editor

PLOS Global Public Health

***********************************************************

Reviewer Comments (if any, and for reference):
